# On topological indices and entropy measures of beryllonitrene network via logarithmic regression model

**DOI:** 10.1038/s41598-024-57601-1

**Published:** 2024-03-26

**Authors:** Guofeng Yu, Muhammad Kamran Siddiqui, Mazhar Hussain, Nazir Hussain, Zohaib Saddique, Fikre Bogale Petros

**Affiliations:** 1 Public Courses Education Department, Anhui Business Vocational College, Hefei, 230031 Anhui China; 2 Department of Mathematics, COMSATS University Islamabad, Lahore Campus, Lahore, Pakistan; 3 School of Chemistry, University of the Punjab, Quaid-i-Azam Campus, Lahore, Pakistan; 4 Department of Mathematics, Addis Ababa University, Addis Ababa, Ethiopia

**Keywords:** Topological indices, Beryllonitrene, Entropy, Regression analysis, Logarithmic model, SPSS, Applied mathematics, Statistics

## Abstract

Chemical graph theory, a subfield of graph theory, is used to investigate chemical substances and their characteristics. Chemical graph analysis sheds light on the connection, symmetry, and reactivity of molecules. It supports chemical property prediction, research of molecular reactions, drug development, and understanding of molecular networks. A crucial part of computational chemistry is chemical graph theory, which helps researchers analyze and manipulate chemical structures using graph algorithms and mathematical models. Beryllonitrene , a compound of interest due to its potential applications in various fields, is examined through the lens of graph theory and mathematical modeling. The study involves the calculation and interpretation of topological indices and graph entropy measures, which provide valuable insights into the structural and energetic properties of Beryllonitrene’s molecular graph. Logarithmic regression models are employed to establish correlations between these indices, entropy, and other relevant molecular attributes. The results contribute to a deeper understanding of Beryllonitrene’s complex characteristics, facilitating its potential applications in diverse scientific and technological domains. In this study, degree-based topological indices $\text{TI}$ are determined, as well as the entropy of graphs based on these $\text{TI}$.

## Introduction

Graph theory is a subfield of mathematics concerned with the study of graphs. A graph is a mathematical structure composed of a collection of objects known as vertices or nodes and a set of connections between these items known as edges. Relationships between distinct items are shown and analyzed using graphs^1^. The vertices of a graph represent items like cities, individuals, or molecules, while the edges reflect the connections or interactions between these entities. Edges can be directed (for a one-way connection) or undirected (for a two-way connection)^2^. Weights can also be added to graph edges to signify the strength or expense of the connections. the totla number of edges incident to a vertex is called the degree of that vertex and denoted by $\S (\tau )$^3^.

Graph theory provides a strong foundation for modeling and understanding complex systems and relationships. It provides tools and approaches for solving problems involving connectivity, optimization, and structure in graphs, and it has a wide range of real-world applications^4^. Topological indices are mathematical descriptors that analyze a molecule’s molecular graph to determine its connectivity and structural characteristics^5^. On the other hand, a compound’s physicochemical qualities are its physical and chemical characteristics that control how it behaves and interacts with other systems^6^. The connection between topological indices and a molecule’s physicochemical characteristics is supported by the idea that molecular structure affects molecular properties. Different topological indices capture distinct molecular structure components, which may affect or correlate with different physical properties^7^.

Topological indices such as the Wiener, Randic, and Zagreb indices reflect a molecule’s size or shape^8,9^. Higher values of these indices generally indicate larger or more complicated molecules, which can be related to qualities such as molecular weight, boiling temperature, or viscosity^10,11^. The topological polar surface area (TPSA) index measures the polar surface area of a molecule. Because of greater polarity, hydrogen bonding capacity, and interactions with solvent molecules, compounds with higher *TPSA* tend to have higher water solubility^12^. The logarithm of the octanol-water partition coefficient (*logP*) is a standard indicator of a compound’s lipophilicity or hydrophobicity. Some topological indices, such as the Balaban index and the connection index, have been discovered to correlate with *logP* values, implying a link between molecular structure and hydrophobic characteristics^13^.

Topological indices can reveal information about a molecule’s chemical reactivity. The Szeged index, for example, or the edge-connectivity index, can be used to predict a compound’s stability or reactivity. Liu et al.^14,15^ analyses of some structural properties of networks. Higher values of these indices may imply stronger chemical stability or resistance^16^. While topological indices can provide useful information about molecular structure and potential correlations with physicochemical features, they can not capture the full complexity of intermolecular interactions^17,18^. Nadeem et al.^19^ discussed the topological aspects of metal-organic structures. Ahmad et al.^20,21^ analysis the theoretical study of energy of phenylene and anthracene. Koam et.al^22^ computed the valency-based topological descriptor for Hexagon Star Networks. Liu et al.^23,24^ compute Hosoya index of some graphs based on connection number.They cannot predict all aspects of compound behavior. Other elements that influence physicochemical qualities include electronic structure, stereochemistry, and intermolecular forces^25^. As a result, a thorough understanding of compound properties frequently necessitates the consideration of many elements in addition to topological indices. Some Topological index are given in Table 1.

**Table 1:** Topological indies $\text{TIs}$ along with their general formulas.

Index	General formula
$Randic\,\ Index$^26^	$\text{R}_{\alpha }\text {(G)}=\sum _{ \tau \varsigma \in E(G)} (\S (\tau )\times \S (\varsigma ))^{\alpha }$
$Atom\,\ Bond\,\ Connectivity\,\ Index$^27,28^	$\text {ABC(G)}=\sum _{ \tau \varsigma \in E(G)}\sqrt{\frac{\S (\tau )+\S (\varsigma )-2}{\S (\tau )\times \S (\varsigma )}}$
$Geometric\,\ Arithmetic\,\ Index$^29^	$\text {GA(G)}=\sum _{ \tau \varsigma \in E(G)}\frac{2\sqrt{\S (\tau )\times \S (\varsigma )}}{\S (\tau )+\S (\varsigma )}$
$First\,\ Zagreb\,\ Index$^30–32^	$\text{M}_\text {1(G)}=\sum _{ \tau \varsigma \in E(G)}{\S (\tau )+\S (\varsigma )}$
$Second\,\ Zagreb\,\ Index$	$\text{M}_\text {2(G)}=\sum _{ \tau \varsigma \in E(G)}{\S (\tau )\times \S (\varsigma )}$
$Harmonic\,\ Zagreb\,\ Index$^33^	$\text {HM(G)}=\sum _{ \tau \varsigma \in E(G)}(\S (\tau )+\S (\varsigma ))^2$
$Forgotton\,\ Index$^13^	$\text {F(G)}=\sum _{ \tau \varsigma \in E(G)}(\S (\tau )^2+\S (\varsigma )^2)$
$First\,\ Redefined\,\ Zagreb\,\ Index$^34^	$\text{ReZG}_\text {1(G)}=\sum _{ \tau \varsigma \in E(G)}\frac{\S (\tau )+\S (\varsigma )}{\S (\tau )\times \S (\varsigma )}$
$Second\,\ Redefined\,\ Zagreb\,\ Index$	$\text{ReZG}_\text {2(G)}=\sum _{ \tau \varsigma \in E(G)}\frac{\S (\tau )\times \S (\varsigma )}{\S (\tau )+\S (\varsigma )}$
$Third\,\ Redefined\,\ Zagreb\,\ Index$	$\text{ReZG}_\text {3(G)}=\sum _{ \tau \varsigma \in E(G)}\bigg ((\S (\tau )\times \S (\varsigma ))(\S (\tau )+\S (\varsigma )\bigg )$

## Topological indices for beryllonitrene $BeN_4$

**Figure 1:**
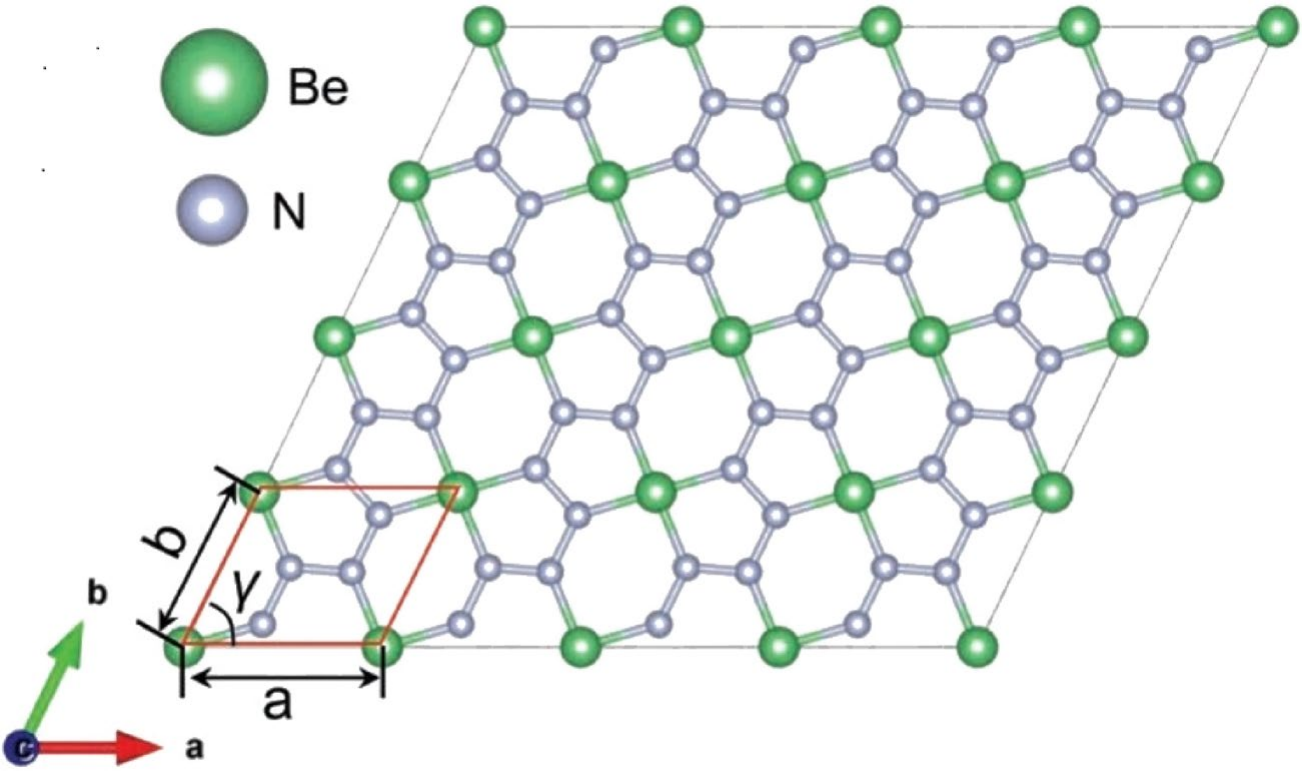
Beryllonitrene $BeN_4$ sheet with unit cell^35^.

The structural organization of the chemical beryllonitrene is distinctive and fascinating. It is made up of a covalently linked network of beryllium (Be) and nitrogen (N) atoms. In a typical beryllonitrene molecule, four nitrogen atoms are connected to each beryllium atom, which forms the core of a tetrahedral coordination^35^. A crystal lattice or molecular network that resembles a three-dimensional honeycomb pattern is produced as a result of this arrangement shown in Fig. 1. Beryllonitrene has unique electrical and mechanical properties due to the alternation of beryllium and nitrogen atoms. Because of its extraordinary stability and electrical conductivity capabilities, beryllonitrene is of interest in a variety of sectors, including materials science and electronics. This is because beryllium, which is lightweight, forms strong covalent bonds with nitrogen^36^.

Let $\text{G}=BeN_4$ be the molecular graph of Beryllonitrene having $5mn+m+n+1$ number of vertices and $8mn-n$ number of edges. The vertex division of the molecular graph is given in Table 2, while the edge division is given in Table 3.

**Table 2:** The vertex division for the chemical graph of beryllonitrene $BeN_4$.

$\S (\tau )$	1	2	3	4
*Frequency*	4	$2m+4n-4$	$4mn-2n$	$mn-m-n+1$

**Table 3:** The edge division for the chemical graph of beryllonitrene $BeN_4$.

$(\S (\tau ),\S (\varsigma ))$	(1, 2)	(1, 3)	(2, 2)	(2, 3)	(3, 3)	(3, 4)
*Frequency*	2	2	$2n-2$	$4m+4n-6$	$4mn-3n$	$4mn-4m-4n+4$

**Figure 2:**
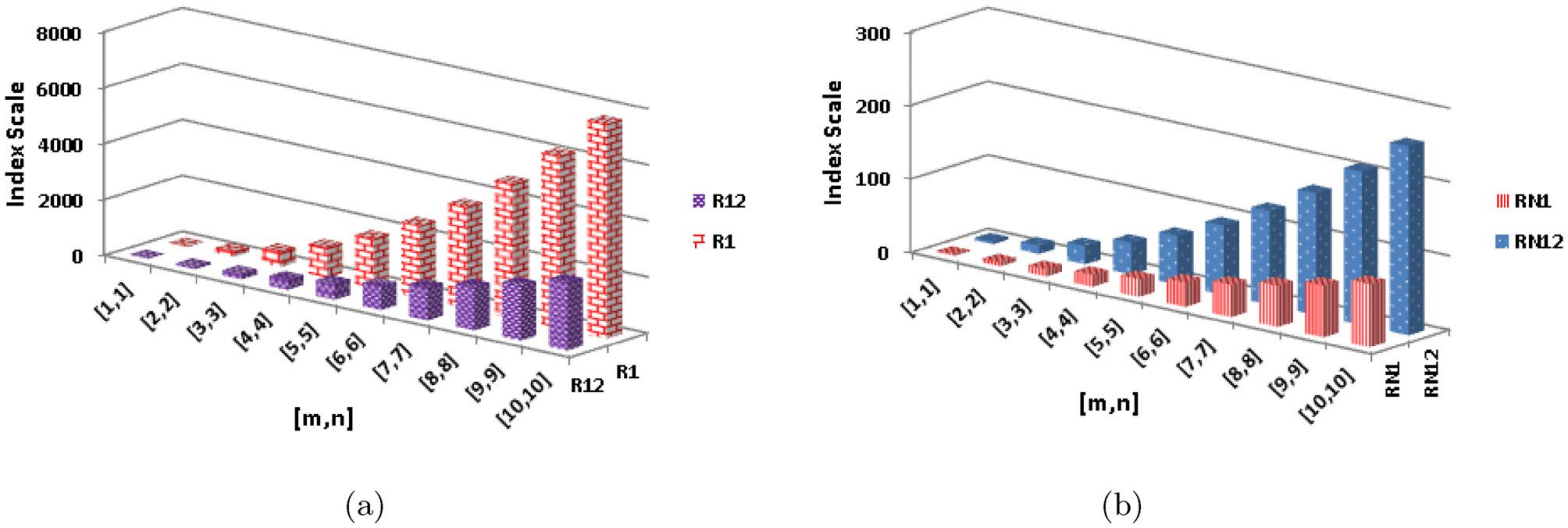
The graphical representation of ${\text{R}}_{1}   ({\text{BeN}}_{4} )$, ${\text{R}}_{{ - 1}} ({\text{BeN}}_{4} )$, ${\text{R}}_{{\frac{1}{2}}} ({\text{BeN}}_{4}$ and ${\text{R}}_{{ - \frac{1}{2}}} ({\text{BeN}}_{4} )$.
- **Atom bond connectivity index**
  $$\begin{aligned}  {\text{ABC}}({\text{BeN}}_{4} ) &=(2)\left( \sqrt{\frac{1+2-2}{1\times 2}}\right) +(2)\left( \sqrt{\frac{1+3-2}{1\times 3}}\right) +(2n-2)\left( \sqrt{\frac{2+2-2}{2\times 2}}\right) +(4m+4n-6)\left( \sqrt{\frac{2+3-2}{2\times 3}}\right) \\&\quad +(4mn-3n)\left( \sqrt{\frac{3+3-2}{3\times 3}}\right) +(4mn-4m-4n+4)\left( \sqrt{\frac{3+4-2}{3\times 4}}\right) \\&=(2)\left( \sqrt{\frac{1}{2}}\right) +(2)\left( \sqrt{\frac{2}{3}}\right) +(2n-2)\left( \sqrt{\frac{2}{4}}\right) +(4m+4n-6)\left( \sqrt{\frac{3}{6}}\right) \\&\quad +(4mn-3n)\left( \sqrt{\frac{4}{9}}\right) +(4mn-4m-4n+4)\left( \sqrt{\frac{5}{12}}\right) \\&=5.2486mn+0.2464m-1.0060n-0.0276 \end{aligned}$$
- **Geometric arithmetic index**
  $$\begin{aligned}  {\text{GA}}({\text{BeN}}_{4} ) &=(2)\left( \frac{2\sqrt{1\times 2}}{1+2}\right) +(2)\left( \frac{2\sqrt{1\times 3}}{1+3}\right) +(2n-2)\left( \frac{2\sqrt{2\times 2}}{2+2}\right) +(4m+4n-6)\left( \frac{2\sqrt{2\times 3}}{2+3}\right) \\&\quad +(4mn-3n)\left( \frac{2\sqrt{3\times 3}}{3+3}\right) +(4mn-4m-4n+4)\left( \frac{2\sqrt{3\times 4}}{3+4}\right) \\&=(2)\left( \frac{2\sqrt{2}}{3}\right) +(2)\left( \frac{2\sqrt{3}}{4}\right) +(2n-2)\left( \frac{2\sqrt{4}}{4}\right) +(4m+4n-6)\left( \frac{2\sqrt{6}}{5}\right) \\&\quad +(4mn-3n)\left( \frac{2\sqrt{9}}{6}\right) +(4mn-4m-4n+4)\left( \frac{2\sqrt{12}}{7}\right) \\&=7.9589mn-0.0793m-2.0397n-0.3021 \end{aligned}$$
- **First zagreb index**
  $$\begin{aligned}  {\text{M}}_{1} ({\text{BeN}}_{4} ) &=(2)(1+2)+(2)(1+3)+(2n-2)(2+2)+(4m+4n-6)(2+3)\\&\quad +(4mn-3n)(3+3)+(4mn-4m-4n+4)(3+4)\\&=(2)(3)+(2)(4)+(2n-2)(4)+(4m+4n-6)(5)+(4mn-3n)(6)+(4mn-4m-4n+4)(7)\\&=52mn-8m-24n+4 \end{aligned}$$
- **Second zagreb index**
  $$\begin{aligned}  {\text{M}}_{2} ({\text{BeN}}_{4} ) &=(2)(1\times 2)+(2)(1\times 3)+(2n-2)(2\times 2)+(4m+4n-6)(2\times 3)\\&\quad +(4mn-3n)(3\times 3)+(4mn-4m-4n+4)(3\times 4)\\&=(2)(2)+(2)(3)+(2n-2)(4)+(4m+4n-6)(6)+(4mn-3n)(9)+(4mn-4m-4n+4)(12)\\&=84mn-24m-52n+14 \end{aligned}$$
  The numerical and graphical representation of ${\text{ABC}}({\text{BeN}}_{4} )$, ${\text{GA}}({\text{BeN}}_{4} )$, ${\text{M}}_{1} ({\text{BeN}}_{4} )$ and ${\text{M}}_{2} ({\text{BeN}}_{4} )$ is shown in Table 5 and Fig. 3, respectively.

- **General randic index**
  **For** $$\alpha =1$$
  $$\begin{aligned} \text{R}_{1}\text {(BeN} _{4} ) &=(2)(1\times 2)+(2)(1\times 3)+(2n-2)(2\times 2)+(4m+4n-6)(2\times 3)\\&\quad +(4mn-3n)(3\times 3)+(4mn-4m-4n+4)(3\times 4)\\&=(2)(2)+(2)(3)+(2n-2)(4)+(4m+4n-6)(6)+(4mn-3n)(9)\\&\quad +(4mn-4m-4n+4)(12)\\&=84mn-24m-52n+14 \end{aligned}$$
  **For** $$\alpha =-1$$
  $$\begin{aligned} \text{R}_{-1}\text {(BeN} _{4} ) &=(2)\left( \frac{1}{1\times 2} \right) +(2)\left( \frac{1}{1\times 3}\right) +(2n-2)\left( \frac{1}{2\times 2}\right) +(4m+4n-6)\left( \frac{1}{2\times 3}\right) \\&\quad +(4mn-3n)\left( \frac{1}{3\times 3}\right) +(4mn-4m-4n+4)\left( \frac{1}{3\times 4}\right) \\&=(2)\left( \frac{1}{2}\right) +(2)\left( \frac{1}{3}\right) +(2n-2)\left( \frac{1}{4}\right) +(4m+4n-6)\left( \frac{1}{6}\right) \\&\quad +(4mn-3n)\left( \frac{1}{9}\right) +(4mn-4m-4n+4)\left( \frac{1}{12}\right) \\&=0.7778mn+0.3333m+0.3889n+0.5 \end{aligned}$$
  **For** $$\alpha =\frac{1}{2}$$
  $$\begin{aligned} \text{R}_{\frac{1}{2}}\text {(BeN} _{4} ) &=(2)(\sqrt{1\times 2})+(2)(\sqrt{1\times 3})+(2n-2)(\sqrt{2\times 2})+(4m+4n-6)(\sqrt{2\times 3})\\&\quad +(4mn-3n)(\sqrt{3\times 3})+(4mn-4m-4n+4)(\sqrt{3\times 4})\\&=(2)(\sqrt{2})+(2)(\sqrt{3})+(2n-2)(\sqrt{4})+(4m+4n-6)(\sqrt{6})\\&\quad +(4mn-3n)(\sqrt{9})+(4mn-4m-4n+4)(\sqrt{12})\\&=25.8564mn-4.0584m-12.0584n+1.4519 \end{aligned}$$
  **For** $$\alpha =-\frac{1}{2}$$
  $$\begin{aligned} \text{R}_{-\frac{1}{2}}\text {(BeN} _{4} ) &=(2)\left( \frac{1}{\sqrt{1\times 2}}\right) +(2)\left( \frac{1}{\sqrt{1\times 3}}\right) +(2n-2)\left( \frac{1}{\sqrt{2\times 2}}\right) +(4m+4n-6)\left( \frac{1}{\sqrt{2\times 3}}\right) \\&\quad +(4mn-3n)\left( \frac{1}{\sqrt{3\times 3}}\right) +(4mn-4m-4n+4)\left( \frac{1}{\sqrt{3\times 4}}\right) \\&=(2)\left( \frac{1}{\sqrt{2}}\right) +(2)\left( \frac{1}{\sqrt{3}}\right) +(2n-2)\left( \frac{1}{\sqrt{4}}\right) +(4m+4n-6)\left( \frac{1}{\sqrt{6}}\right) \\&\quad +(4mn-3n)\left( \frac{1}{\sqrt{9}}\right) +(4mn-4m-4n+4)\left( \frac{1}{\sqrt{12}}\right) \\&=2.4880mn+0.4783m+0.1449n+0.2741 \end{aligned}$$
  The numerical and graphical representation of $\text{R}_{1}\text {(BeN} _{4} )$, ${\text{R}}_{{ - 1}} ({\text{BeN}}_{4} )$, $\text{R}_{\frac{1}{2}}\text {(BeN} _{4} )$ and $\text{R}_{-\frac{1}{2}}\text {(BeN} _{4} )$ is shown in Table 4 and Fig. 2, respectively.

**Table 4:** The numerical representation of $\text{R}_{1}\text {(BeN} _{4} )$, $\text{R}_{-1}\text {(BeN} _{4} )$, $\text{R}_{\frac{1}{2}}\text {(BeN} _{4} )$ and $\text{R}_{-\frac{1}{2}}\text {(BeN} _{4} )$.

[*m*, *n*]	[1, 1]	[2, 2]	[3, 3]	[4, 4]	[5, 5]	[6, 6]	[7, 7]	[8, 8]	[9, 9]	[10, 10]
${\text{R}}_{1} ({\text{BeN}}_{4} )$	22	198	542	1054	1734	2582	3598	4782	6134	7654
${\text{R}}_{1} ({\text{BeN}}_{4} )$	2	5.06	9.67	15.83	23.56	32.83	43.68	56.06	70.00	85.50
${\text{R}}_{{\frac{1}{2}}} ({\text{BeN}}_{4}$	11.19	72.64	185.81	350.69	567.28	835.58	1155.59	1527.33	1950.77	2425.92
${\text{R}}_{{ - \frac{1}{2}}} ({\text{BeN}}_{4} )$	3.39	11.47	24.54	42.57	65.59	93.58	126.55	164.49	207.41	255.31

**Table 5:** The numerical representation of ${\text{ABC}}({\text{BeN}}_{4} )$, ${\text{GA}}({\text{BeN}}_{4} )$, ${\text{M}}_{1} ({\text{BeN}}_{4} )$ and ${\text{M}}_{2} ({\text{BeN}}_{4} )$.

[*m*, *n*]	[1, 1]	[2, 2]	[3, 3]	[4, 4]	[5, 5]	[6, 6]	[7, 7]	[8, 8]	[9, 9]	[10, 10]
${\text{ABC}}({\text{BeN}}_{4} )$	4.46	19.44	44.93	80.91	127.38	184.36	251.83	329.80	418.27	517.23
${\text{GA}}({\text{BeN}}_{4} )$	5.53	27.29	64.97	118.56	188.07	273.50	374.85	492.11	625.29	774.39
${\text{M}}_{1} ({\text{BeN}}_{4} )$	24	148	376	708	1144	1684	2328	3076	3928	4884
${\text{M}}_{2} ({\text{BeN}}_{4} )$	22	198	542	1054	1734	2582	3598	4782	6134	7654

**Figure 3:**
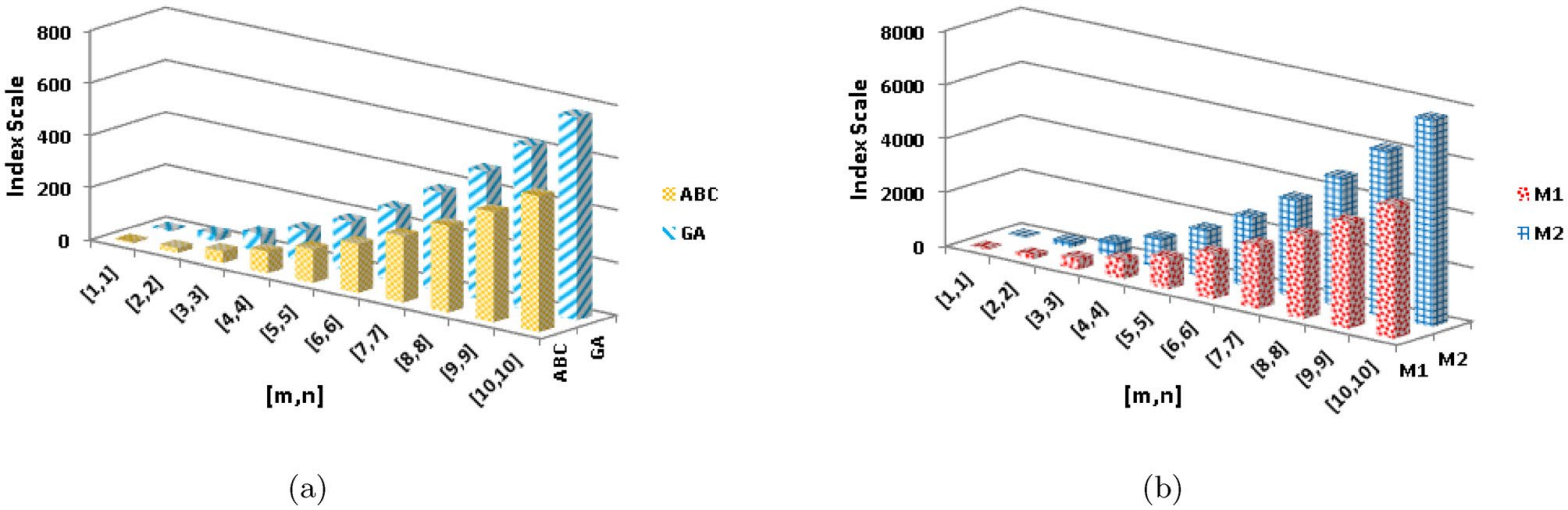
The graphical representation of ${\text{ABC}}({\text{BeN}}_{4} )$, ${\text{GA}}({\text{BeN}}_{4} )$, ${\text{M}}_{1} ({\text{BeN}}_{4} )$ and ${\text{M}}_{2} ({\text{BeN}}_{4} )$.
- **Harmonic zagreb index **
  $$\begin{aligned}  {\text{HM}}({\text{BeN}}_{4} ) &=(2)(1+2)^2+(2)(1+3)^2+(2n-2)(2+2)^2+(4m+4n-6)(2+3)^2\\&\quad +(4mn-3n)(3+3)^2+(4mn-4m-4n+4)(3+4)^2\\&=(2)(3)^2+(2)(4)^2+(2n-2)(4)^2+(4m+4n-6)(5)^2+(4mn-3n)(6)^2+(4mn-4m-4n+4)(7)^2\\&=(2)(9)+(2)(16)+(2n-2)(16)+(4m+4n-6)(25)+(4mn-3n)(36)+(4mn-4m-4n+4)(49)\\&=340mn-96m-208n+64 \end{aligned}$$
- **Forgotton index**
  $$\begin{aligned}  {\text{F}}({\text{BeN}}_{4} ) &=(2)(1^2+2^2)+(2)(1^2+3^2)+(2n-2)(2^2+2^2)+(4m+4n-6)(2^2+3^2)\\&\quad +(4mn-3n)(3^2+3^2)+(4mn-4m-4n+4)(3^2+4^2)\\&=(2)(1+4)+(2)(1+9)+(2n-2)(4+4)+(4m+4n-6)(4+9)+(4mn-3n)(9+9)\\&\quad +(4mn-4m-4n+4)(9+16)\\&=(2)(4)+(2)(10)+(2n-2)(8)+(4m+4n-6)(13)+(4mn-3n)(18)+(4mn-4m-4n+4)(25)\\&=172mn-48m-104n+36 \end{aligned}$$
- **Augmented zagreb index**
  $$\begin{aligned}  {\text{AZI}}({\text{BeN}}_{4} ) &=(2)\left( \frac{1\times 2}{1+2-2}\right) ^3+(2)\left( \frac{1\times 3}{1+3-2}\right) ^3+(2n-2)\left( \frac{2\times 2}{2+2-2}\right) ^3+(4m+4n-6)\left( \frac{2\times 3}{2+3-2}\right) ^3\\&\quad +(4mn-3n)\left( \frac{3\times 3}{3+3-2}\right) ^3+(4mn-4m-4n+4)\left( \frac{3\times 4}{3+4-2}\right) ^3\\&=(2)(\frac{2}{1})+(2)\left( \frac{3}{2}\right) +(2n-2)\left( \frac{4}{2}\right) +(4m+4n-6)\left( \frac{6}{3}\right) \\&\quad +(4mn-3n)\left( \frac{9}{4}\right) +(4mn-4m-4n+4)\left( \frac{12}{5}\right) \\&=100.8585mn-23.2960m-52.8585n+14.0460 \end{aligned}$$
- **First redefined zagreb index**
  $$\begin{aligned}  {\text{ReZG}}_{1} ({\text{BeN}}_{4} ) &=(2)(\frac{1+2}{1\times 2})+(2)(\frac{1+3}{1\times 3})+(2n-2)(\frac{2+2}{2\times 2})+(4m+4n-6)(\frac{2+3}{2\times 3})\\&\quad +(4mn-3n)(\frac{3+3}{3\times 3})+(4mn-4m-4n+4)(\frac{3+4}{3\times 4})\\&=(2)(\frac{3}{2})+(2)(\frac{4}{3})+(2n-2)(\frac{4}{4})+(4m+4n-6)(\frac{5}{6})\\&\quad +(4mn-3n)(\frac{6}{9})+(4mn-4m-4n+4)(\frac{7}{12})\\&=5mn+m+0.3333n+1 \end{aligned}$$
  The numerical and graphical representation of ${\text{HM}}({\text{BeN}}_{4} )$, ${\text{F}}({\text{BeN}}_{4} )$, ${\text{AZI}}({\text{BeN}}_{4} )$ and ${\text{ReZG}}_{1} ({\text{BeN}}_{4} )$ is shown in Table 6 and Fig. 4, respectively.

**Table 6:** The numerical representation of ${\text{HM}}({\text{BeN}}_{4} )$, ${\text{F}}({\text{BeN}}_{4} )$, ${\text{AZI}}({\text{BeN}}_{4} )$ and ${\text{ReZG}}_{1} ({\text{BeN}}_{4} )$.

[*m*, *n*]	[1, 1]	[2, 2]	[3, 3]	[4, 4]	[5, 5]	[6, 6]	[7, 7]	[8, 8]	[9, 9]	[10, 10]
${\text{HM}}({\text{BeN}}_{4} )$	100	816	2212	4288	7044	10480	14596	19392	24868	31024
${\text{F}}({\text{BeN}}_{4} )$	56	420	1128	2180	3576	5316	7400	9828	12600	15716
${\text{AZI}}({\text{BeN}}_{4} )$	38.75	265.17	693.30	1323.16	2154.73	3188.02	4423.03	5859.75	7498.19	9338.35
${\text{ReZG}}_{1} ({\text{BeN}}_{4} )$	7.33	23.66	49.99	86.33	132.66	188.99	255.33	331.66	417.99	514.33

**Figure 4:**
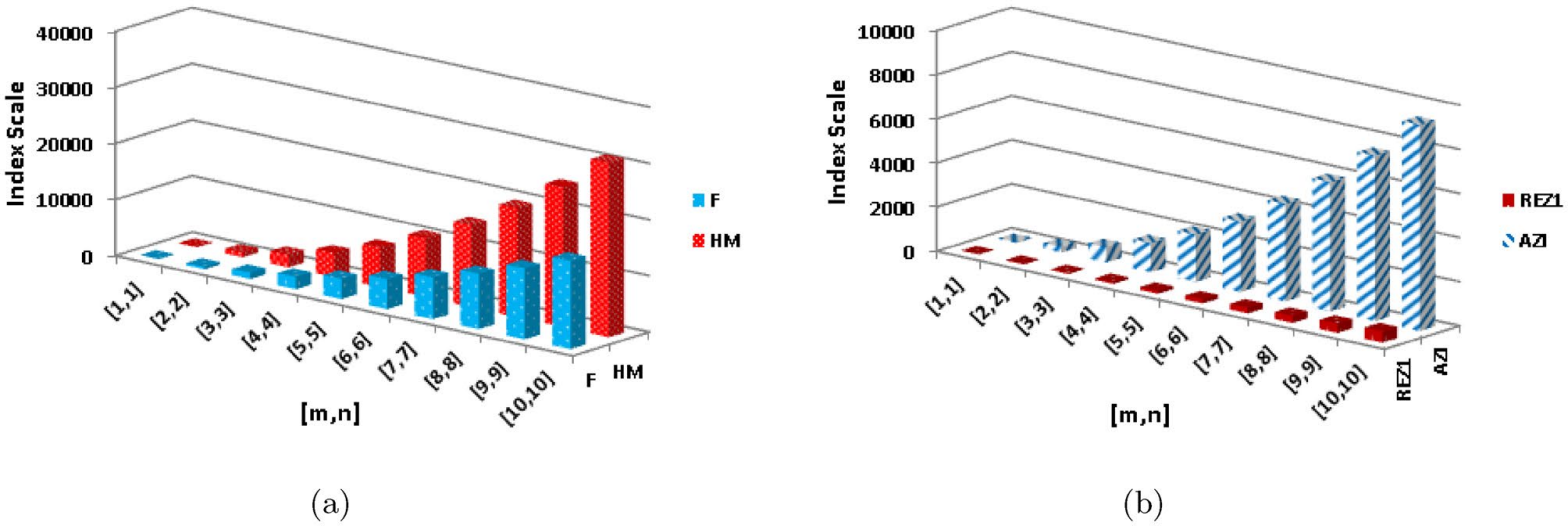
The graphical representation of ${\text{HM}}({\text{BeN}}_{4} )$, ${\text{F}}({\text{BeN}}_{4} )$, ${\text{AZI}}({\text{BeN}}_{4} )$ and ${\text{ReZG}}_{1} ({\text{BeN}}_{4} )$.
- **Second redefined zagreb index**
  $$\begin{aligned}  {\text{ReZG}}_{2} ({\text{BeN}}_{4} ) &=(2)(\frac{1\times 2}{1+2})+(2)(\frac{1\times 3}{1+3})+(2n-2)(\frac{2\times 2}{2+2})+(4m+4n-6)(\frac{2\times 3}{2+3})\\&\quad +(4mn-3n)(\frac{3\times 3}{3+3})+(4mn-4m-4n+4)(\frac{3\times 4}{3+4})\\&=(2)(\frac{2}{3})+(2)(\frac{3}{4})+(2n-2)(\frac{4}{4})+(4m+4n-6)(\frac{6}{5})\\&\quad +(4mn-3n)(\frac{9}{6})+(4mn-4m-4n+4)(\frac{12}{7})\\&=12.8571mn-2.0571m-6.0571n+0.4905 \end{aligned}$$
- **Third redefined zagreb index**
  $$\begin{aligned}  {\text{ReZG}}_{3} ({\text{BeN}}_{4} ) &=(2)((1+2)(1\times 2))+(2)((1+3)(1\times 3))+(2n-2)((2+2)(2\times 2))\\{} & {} +(4m+4n-6)((2+3)(2\times 3))\\{} & {} +(4mn-3n)((3+3)(3\times 3))+(4mn-4m-4n+4)((3+4)(3\times 4))\\{} & {} =(2)(3\times 2)+(2)(4\times 3)+(2n-2)(4\times 4)+(4m+4n-6)(5\times 6)\\{} & {} +(4mn-3n)(6\times 9)+(4mn-4m-4n+4)(7\times 12)\\{} & {} =(2)(6)+(2)(12)+(2n-2)(16)+(4m+4n-6)(30)\\{} & {} +(4mn-3n)(54)+(4mn-4m-4n+4)(84)\\{} & {} =544mn-208m-392n+152 \end{aligned}$$
  The numerical and graphical representation of ${\text{ReZG}}_{2} ({\text{BeN}}_{4} )$ and ${\text{ReZG}}_{3} ({\text{BeN}}_{4} )$ is shown in Table 7 and Fig. 5, respectively.

**Table 7:** The numerical representation of ${\text{ReZG}}_{2} ({\text{BeN}}_{4} )$ and ${\text{ReZG}}_{3} ({\text{BeN}}_{4} )$.

[*m*, *n*]	[1, 1]	[2, 2]	[3, 3]	[4, 4]	[5, 5]	[6, 6]	[7, 7]	[8, 8]	[9, 9]	[10, 10]
${\text{ReZG}}_{2} ({\text{BeN}}_{4} )$	5.23	35.69	91.86	173.74	281.34	414.66	573.68	758.43	968.88	1205.05
${\text{ReZG}}_{3} ({\text{BeN}}_{4} )$	96	1128	3248	6456	10752	16136	22608	30168	38816	48552

**Figure 5:**
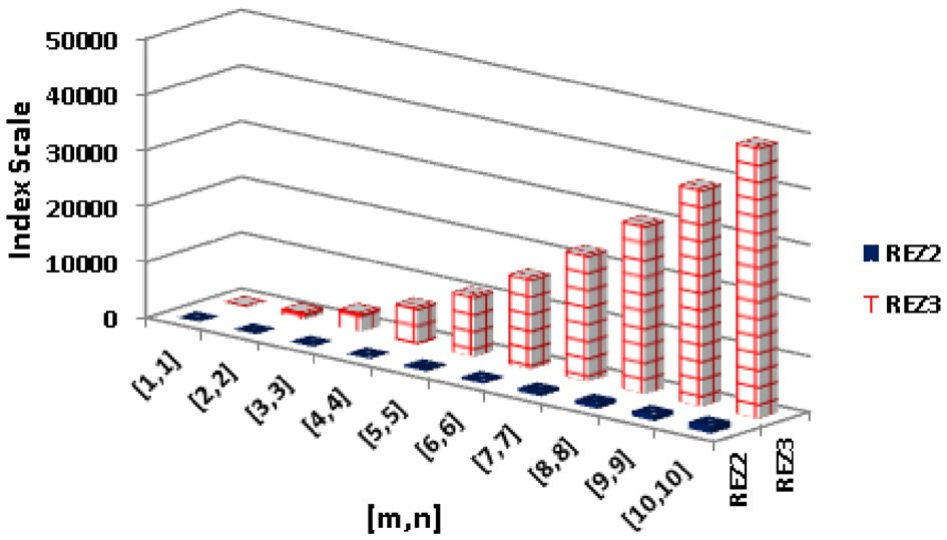
The numerical and graphical representation of ${\text{ReZG}}_{2} ({\text{BeN}}_{4} )$ and ${\text{ReZG}}_{3} ({\text{BeN}}_{4} )$.

## Graph entropy

Entropy is the measurement of disorders of a system while the measurement of unpredictability of information content or the measurement of uncertainty of a system also called the entropy of a system, the concept was introduce in 1948^37^. The concept of graph entropy was applied in chemistry, biology, and other sciences^38^. There are different types of graphs for measuring entropy, for exploring the network the degree power is most significant.

1 $$\begin{aligned} ENT_{I}&=-\sum _{i=1}^{m}\Theta _{i}{\frac{I(\rho _{i}\varrho _{i})}{(\varpi _{d})}}\log {\frac{I(\rho _{i}\varrho _{i})}{(\varpi _{d})}} =\log ({\varpi _{d}})-\frac{1}{(\varpi _{d})}\sum _{i=1}^{m}\Theta _{i}{I(\rho _{i}\varrho _{i})}\log {I(\rho _{i}\varrho _{i})} \end{aligned}$$

where $\varpi _{d}=\sum _{i=1}^{m}\Theta _{i}{I(\rho _{i}\varrho _{i})}$ is topological index $\Theta _{i}$ is frequency *m* is number of edges $I(\rho \varrho )$ is the weight of the edge $\rho \varrho$ see^37^. By using Tables 1 and 3 and Eq. (1), we have following formulas and their calculation.

In chemistry and related sciences, topological indices are mathematical descriptors that describe the topology of molecular structures. In relation to these indices, entropy may be defined as the degree of randomness or disorder in the distribution of specific structural characteristics. The calculation of entropy using topological indices in the context of molecular structures can offer several benefits.

**Figure 6:**
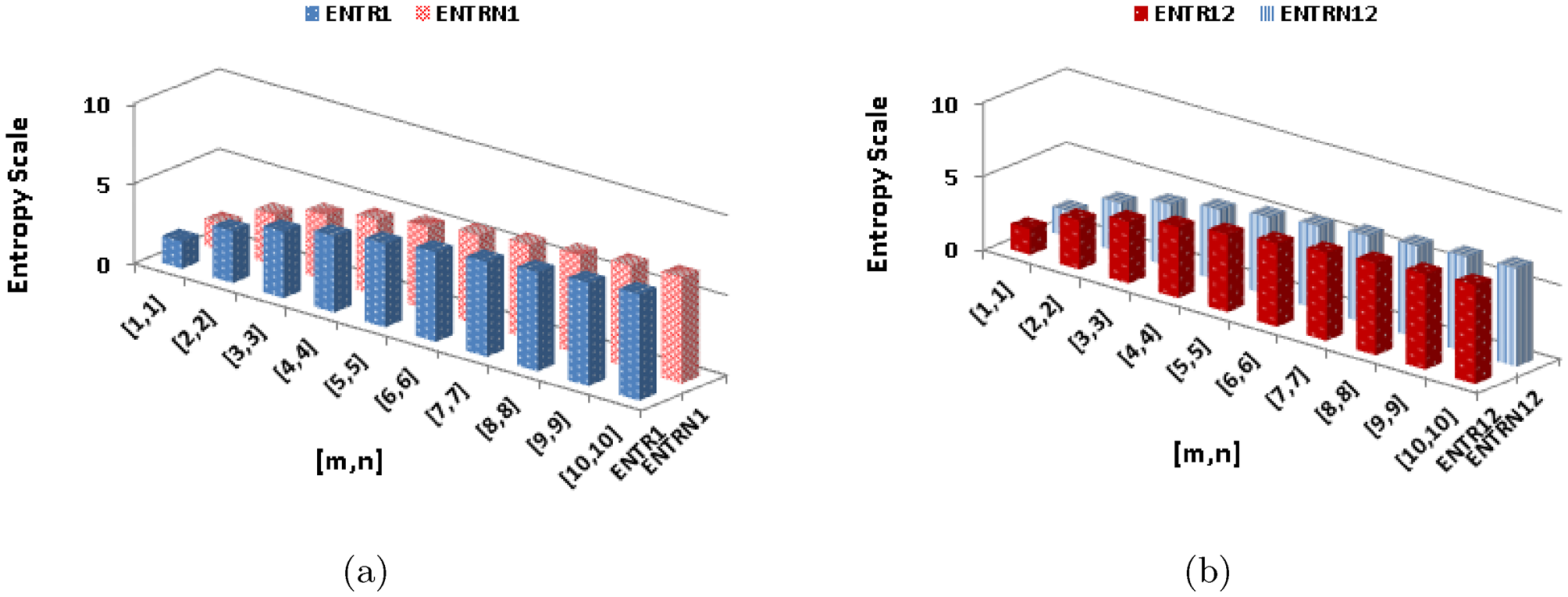
The graphical representation of ${\text{ENT}}_{{ {\text{R}}_{1}   ({\text{BeN}}_{4} )}}$, ${\text{ENT}}_{{{\text{R}}_{{ - 1}} ({\text{BeN}}_{4} )}}$, ${\text{ENT}}_{{{\text{R}}_{{\frac{1}{2}}} ({\text{BeN}}_{4} )}}$ and ${\text{ENT}}_{{{\text{R}}_{{ - \frac{1}{2}}} ({\text{BeN}}_{4} )}}$.
- **Atom bond connectivity entropy**
  $$\begin{aligned}  {\text{ENT}}_{{{\text{ABC}}({\text{BeN}}_{4} )}} &=\log (ABC)-{\frac{1}{(ABC)}}\sum _{i=1}^{6}\Theta {\sqrt{\frac{\rho +\varrho -2}{\rho \times \varrho }}}\log _{2}{\sqrt{\frac{\rho +\varrho -2}{\rho \times \varrho }}}\\ ENT_{ABC}&=\log (5.2486mn+0.2464m-1.0060n-0.0276)-\frac{(2)\log (\sqrt{\frac{1}{2}})^{\sqrt{\frac{1}{2}}}}{5.2486mn+0.2464m-1.0060n-0.0276}\\&\quad -\frac{(2)\log (\sqrt{\frac{2}{3}})^{\sqrt{\frac{2}{3}}}}{5.2486mn+0.2464m-1.0060n-0.0276}-\frac{(2n-2)\log (\sqrt{\frac{2}{4}})^{\sqrt{\frac{2}{4}}}}{5.2486mn+0.2464m-1.0060n-0.0276}\\&\quad -\frac{(4m+4n-6)\log (\sqrt{\frac{3}{6}})^{\sqrt{\frac{3}{6}}}}{5.2486mn+0.2464m-1.0060n-0.0276}-\frac{(4mn-3n)\log (\sqrt{\frac{4}{9}})^{\sqrt{\frac{4}{9}}}}{5.2486mn+0.2464m-1.0060n-0.0276}\\&\quad -\frac{(4mn-4m-4n+4)\log (\sqrt{\frac{5}{12}})^{\sqrt{\frac{5}{12}}}}{5.2486mn+0.2464m-1.0060n-0.0276}\\ \end{aligned}$$
- **Geometric arithmetic entropy**
  $$\begin{aligned}  {\text{ENT}}_{{{\text{GA}}({\text{BeN}}_{4} )}} &=\log (GA)-{\frac{1}{(GA)}}\sum _{i=1}^{6}\Theta {\frac{2\sqrt{\rho \times \varrho }}{\rho +\varrho }}\log _{2}{\frac{2\sqrt{\rho \times \varrho }}{\rho +\varrho }}\\&=\log (7.9589mn-0.0793m-2.0397n-0.3021)-\frac{(2)\log (\frac{2\sqrt{2}}{3})^{\frac{2\sqrt{2}}{3}}}{7.9589mn-0.0793m-2.0397n-0.3021}\\&\quad -\frac{(2)\log (\frac{2\sqrt{3}}{4})^{\frac{2\sqrt{3}}{4}}}{7.9589mn-0.0793m-2.0397n-0.3021}-\frac{(2n-2)\log (\frac{2\sqrt{4}}{4})^{\frac{2\sqrt{4}}{4}}}{7.9589mn-0.0793m-2.0397n-0.3021}\\&\quad -\frac{(4m+4n-6)\log (\frac{2\sqrt{6}}{5})^{\frac{2\sqrt{6}}{5}}}{7.9589mn-0.0793m-2.0397n-0.3021}-\frac{(4mn-3n)\log (\frac{2\sqrt{9}}{6})^{\frac{2\sqrt{9}}{6}}}{7.9589mn-0.0793m-2.0397n-0.3021}\\&\quad -\frac{(4mn-4m-4n+4)\log (\frac{2\sqrt{12}}{7})^{\frac{2\sqrt{12}}{7}}}{7.9589mn-0.0793m-2.0397n-0.3021}\\ \end{aligned}$$
- **First zagreb entropy**
  $$\begin{aligned}  {\text{ENT}}_{{{\text{M}}_{1} ({\text{BeN}}_{4} )}}  &=\log (M_{1})-{\frac{1}{(M_{1})}}\sum _{i=1}^{6}\Theta {(\rho +\varrho )}\log _{2}{(\rho +\varrho )}\\&=\log (52mn-8m-24n+4)-\frac{(2)\log (3)^3}{52mn-8m-24n+4}-\frac{(2)\log (4)^4}{52mn-8m-24n+4}\\&\quad -\frac{(2n-2)\log (4)^4}{52mn-8m-24n+4}-\frac{(4m+4n-6)\log (5)^5}{52mn-8m-24n+4}-\frac{(4mn-3n)\log (6)^6}{52mn-8m-24n+4}\\&\quad -\frac{(4mn-4m-4n+4)\log (7)^7}{52mn-8m-24n+4}\\ \end{aligned}$$
- **Second zagreb entropy**
  $$\begin{aligned} {\text{ENT}}_{{{\text{M}}_{2} ({\text{BeN}}_{4} )}}&=\log (M_{2})-{\frac{1}{(M_{2})}}\sum _{i=1}^{6}\Theta {(\rho \times \varrho )}\log _{2}{(\rho \times \varrho )}\\&=\log (84mn-24m-52n+14)-\frac{(2)\log (2)^2}{84mn-24m-52n+14}-\frac{(2)\log (3)^3}{84mn-24m-52n+14}\\&\quad -\frac{(2n-2)\log (4)^4}{84mn-24m-52n+14}-\frac{(4m+4n-6)\log (6)^6}{84mn-24m-52n+14}-\frac{(4mn-3n)\log (9)^9}{84mn-24m-52n+14}\\&\quad -\frac{(4mn-4m-4n+4)\log (12)^{12}}{84mn-24m-52n+14}\\ \end{aligned}$$
  The numerical and graphical representation of ${\text{ENT}}_{{{\text{ABC}}({\text{BeN}}_{4} )}}$, ${\text{ENT}}_{{{\text{GA}}({\text{BeN}}_{4} )}}$, ${\text{ENT}}_{{{\text{M}}_{1} ({\text{BeN}}_{4} )}}$ and ${\text{ENT}}_{{{\text{M}}_{2} ({\text{BeN}}_{4} )}}$ is shown in Table 9 and Fig. 7, respectively.

- The structural diversity of molecular compounds can be quantitatively evaluated using entropy measures that are derived from topological indices. Greater structural diversity may be indicated by higher entropy values, which would add to a more varied chemical space.
- Entropy measurements are correlated with a number of molecular properties, both chemical and physical. Properties like solubility, boiling points, and reaction rates can be predicted by using topological indices in entropy calculations.
- Entropy makes it possible to compare various molecular sets or chemical databases according to the structural diversity of each set. Entropy values can be used by researchers to rank or screen compounds for additional testing.

- **Randic entropy**
  **For** $$\alpha =1$$
  $$\begin{aligned}   {\text{ENT}}_{{ {\text{R}}_{1}   ({\text{BeN}}_{4} )}}  &=\log (R_{1})-{\frac{1}{(R_{1})}}\sum _{i=1}^{6}\Theta {(\rho \times \varrho )}\log _{2}{(\rho \times \varrho )}\\&=\log (84mn-24m-52n+14)-\frac{(2)\log (2)^2}{84mn-24m-52n+14}-\frac{(2)\log (3)^3}{84mn-24m-52n+14}\\&\quad -\frac{(2n-2)\log (4)^4}{84mn-24m-52n+14}-\frac{(4m+4n-6)\log (6)^6}{84mn-24m-52n+14}-\frac{(4mn-3n)\log (9)^9}{84mn-24m-52n+14}\\&\quad -\frac{(4mn-4m-4n+4)\log (12)^{12}}{84mn-24m-52n+14}\\ \end{aligned}$$
  **For** $$\alpha =-1$$
  $$\begin{aligned}  {\text{ENT}}_{{{\text{R}}_{{ - 1}} ({\text{BeN}}_{4} )}}  &=\log (R_{-1})-{\frac{1}{(R_{-1})}}\sum _{i=1}^{6}\Theta {\frac{1}{(\rho \times \varrho )}}\log _{2}{\frac{1}{(\rho \times \varrho )}}\\&=\log (0.7778mn+0.3333m+0.3889n+0.5)-\frac{(2)\log (\frac{1}{2})^{\frac{1}{2}}}{0.7778mn+0.3333m+0.3889n+0.5}\\&\quad -\frac{(2)\log (\frac{1}{3})^{\frac{1}{3}}}{0.7778mn+0.3333m+0.3889n+0.5}-\frac{(2n-2)\log (\frac{1}{4})^{\frac{1}{4}}}{0.7778mn+0.3333m+0.3889n+0.5}\\&\quad -\frac{(4m+4n-6)\log (\frac{1}{6})^{\frac{1}{6}}}{0.7778mn+0.3333m+0.3889n+0.5}-\frac{(4mn-3n)\log (\frac{1}{9})^{\frac{1}{9}}}{0.7778mn+0.3333m+0.3889n+0.5}\\&\quad -\frac{(4mn-4m-4n+4)\log (\frac{1}{12})^{\frac{1}{12}}}{0.7778mn+0.3333m+0.3889n+0.5}\\ \end{aligned}$$
  **For** $$\alpha =\frac{1}{2}$$
  $$\begin{aligned}  {\text{ENT}}_{{{\text{R}}_{{\frac{1}{2}}} ({\text{BeN}}_{4} )}}  &=\log (R_{\frac{1}{2}})-{\frac{1}{(R_{\frac{1}{2}})}}\sum _{i=1}^{6}\Theta {\sqrt{(\rho \times \varrho )}}\log _{2}{\sqrt{(\rho \times \varrho )}}\\&=\log (25.8564mn-4.0584m-12.0584n+1.4519)-\frac{(2)\log (\sqrt{2})^{\sqrt{2}}}{25.8564mn-4.0584m-12.0584n+1.4519}\\&\quad -\frac{(2)\log (\sqrt{3})^{\sqrt{3}}}{25.8564mn-4.0584m-12.0584n+1.4519}-\frac{(2n-2)\log (\sqrt{4})^{\sqrt{4}}}{25.8564mn-4.0584m-12.0584n+1.4519}\\&\quad -\frac{(4m+4n-6)\log (\sqrt{6})^{\sqrt{6}}}{25.8564mn-4.0584m-12.0584n+1.4519}-\frac{(4mn-3n)\log (\sqrt{9})^{\sqrt{9}}}{25.8564mn-4.0584m-12.0584n+1.4519}\\&\quad -\frac{(4mn-4m-4n+4)\log (\sqrt{12})^{\sqrt{12}}}{25.8564mn-4.0584m-12.0584n+1.4519}\\ \end{aligned}$$
  **For** $$\alpha =\frac{1}{2}$$
  $$\begin{aligned}  {\text{ENT}}_{{{\text{R}}_{{ - \frac{1}{2}}} ({\text{BeN}}_{4} )}}  &=\log (R_{-\frac{1}{2}})-{\frac{1}{(R_{-\frac{1}{2}})}}\sum _{i=1}^{6}\Theta {\frac{1}{\sqrt{(\rho \times \varrho )}}}\log _{2}{\frac{1}{\sqrt{(\rho \times \varrho )}}}\\&=\log (2.4880mn+0.4783m+0.1449n+0.2741)-\frac{(2)\log (\frac{1}{\sqrt{2}})^{\frac{1}{\sqrt{2}}}}{2.4880mn+0.4783m+0.1449n+0.2741}\\&\quad -\frac{(2)\log (\frac{1}{\sqrt{3}})^{\frac{1}{\sqrt{3}}}}{2.4880mn+0.4783m+0.1449n+0.2741}-\frac{(2n-2)\log (\frac{1}{\sqrt{4}})^{\frac{1}{\sqrt{4}}}}{2.4880mn+0.4783m+0.1449n+0.2741}\\&\quad -\frac{(4m+4n-6)\log (\frac{1}{\sqrt{6}})^{\frac{1}{\sqrt{6}}}}{2.4880mn+0.4783m+0.1449n+0.2741}-\frac{(4mn-3n)\log (\frac{1}{\sqrt{9}})^{\frac{1}{\sqrt{9}}}}{2.4880mn+0.4783m+0.1449n+0.2741}\\&\quad -\frac{(4mn-4m-4n+4)\log (\frac{1}{\sqrt{12}})^{\frac{1}{\sqrt{12}}}}{2.4880mn+0.4783m+0.1449n+0.2741}\\ \end{aligned}$$
  The numerical and graphical representation of ${\text{ENT}}_{{ {\text{R}}_{1}   ({\text{BeN}}_{4} )}}$, ${\text{ENT}}_{{{\text{R}}_{{ - 1}} ({\text{BeN}}_{4} )}}$, ${\text{ENT}}_{{{\text{R}}_{{\frac{1}{2}}} ({\text{BeN}}_{4} )}}$ and ${\text{ENT}}_{{{\text{R}}_{{ - \frac{1}{2}}} ({\text{BeN}}_{4} )}}$ is shown in Table 8 and Fig. 6, respectively.

**Table 8:** The numerical representation of ${\text{ENT}}_{{ {\text{R}}_{1}   ({\text{BeN}}_{4} )}}$, ${\text{ENT}}_{{{\text{R}}_{{ - 1}} ({\text{BeN}}_{4} )}}$, ${\text{ENT}}_{{{\text{R}}_{{\frac{1}{2}}} ({\text{BeN}}_{4} )}}$ and ${\text{ENT}}_{{{\text{R}}_{{ - \frac{1}{2}}} ({\text{BeN}}_{4} )}}$.

[*m*, *n*]	[1, 1]	[2, 2]	[3, 3]	[4, 4]	[5, 5]	[6, 6]	[7, 7]	[8, 8]	[9, 9]	[10, 10]
${\text{ENT}}_{{ {\text{R}}_{1} ({\text{BeN}}_{4} )}}$	1.688	3.2406	4.1241	4.7356	5.2034	5.5824	5.9008	6.1755	6.4169	6.6324
${\text{ENT}}_{{ {\text{R}}_{1} ({\text{BeN}}_{4} )}}$	1.7045	3.1804	4.0644	4.6847	5.1603	5.5453	5.8685	6.1469	6.3913	6.6092
${\text{ENT}}_{{{\text{R}}_{{\frac{1}{2}}} ({\text{BeN}}_{4} )}}$	1.7653	3.3056	4.17045	4.7723	5.2343	5.6093	5.925	6.1976	6.4374	6.6516
${\text{ENT}}_{{{\text{R}}_{{ - \frac{1}{2}}} ({\text{BeN}}_{4} )}}$	1.7676	3.2975	4.1628	4.766	5.229	5.6048	5.9211	6.1942	6.4344	6.6488

**Table 9:** The numerical representation of ${\text{ENT}}_{{{\text{ABC}}({\text{BeN}}_{4} )}}$, ${\text{ENT}}_{{{\text{GA}}({\text{BeN}}_{4} )}}$, ${\text{ENT}}_{{{\text{M}}_{1} ({\text{BeN}}_{4} )}}$ and ${\text{ENT}}_{{{\text{M}}_{2} ({\text{BeN}}_{4} )}}$.

[*m*, *n*]	[1, 1]	[2, 2]	[3, 3]	[4, 4]	[5, 5]	[6, 6]	[7, 7]	[8, 8]	[9, 9]	[10, 10]
${\text{ENT}}_{{{\text{ABC}}({\text{BeN}}_{4} )}}$	1.7893	3.3304	4.1884	4.7865	5.2462	5.6196	5.9342	6.206	6.4451	6.6588
${\text{ENT}}_{{{\text{GA}}({\text{BeN}}_{4} )}}$	1.7838	3.3287	4.1875	4.7859	5.2458	5.6194	5.934	6.2058	6.445	6.65873
${\text{ENT}}_{{{\text{M}}_{1} ({\text{BeN}}_{4} )}}$	1.7707	3.3098	4.1724	4.7734	5.235	5.6098	5.9253	6.1978	6.4376	6.6516
${\text{ENT}}_{{{\text{M}}_{2} ({\text{BeN}}_{4} )}}$	1.688	3.2406	4.1241	4.7356	5.2034	5.5824	5.9008	6.1755	6.4169	6.6324

**Figure 7:**
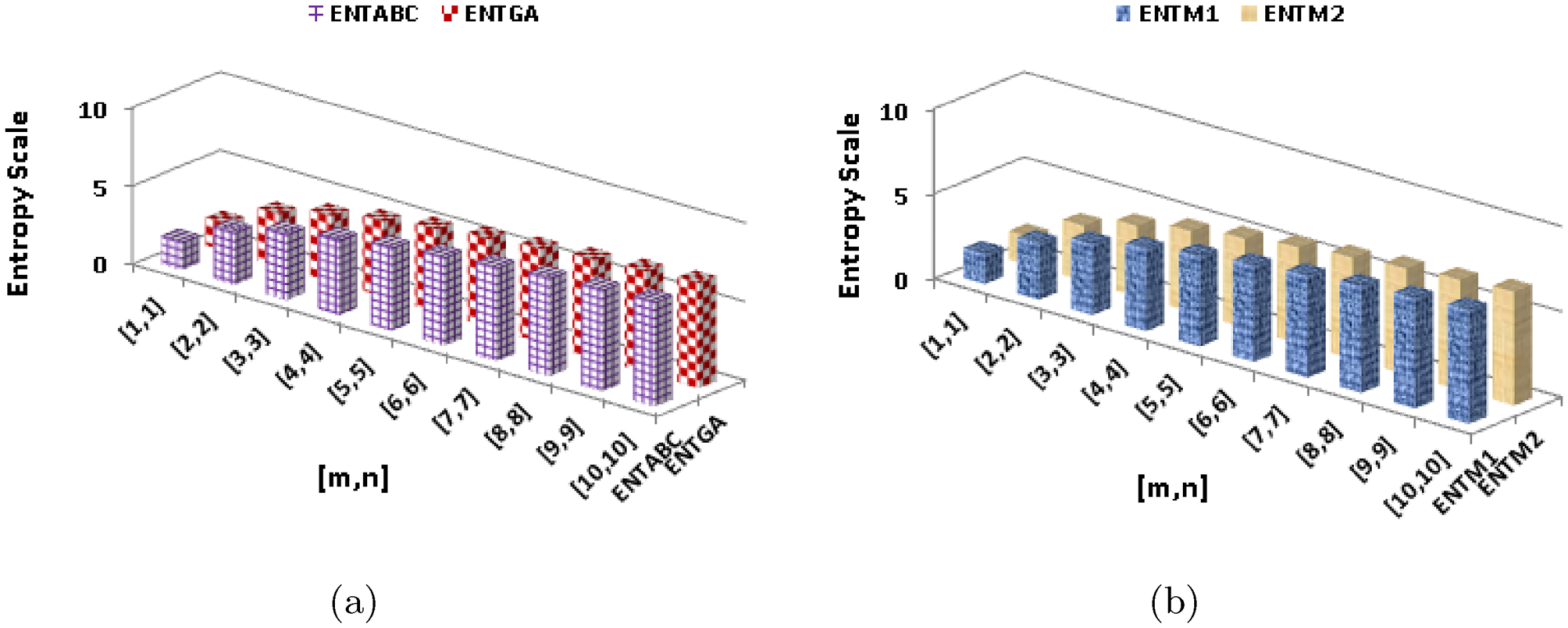
The graphical representation of ${\text{ENT}}_{{{\text{ABC}}({\text{BeN}}_{4} )}}$, ${\text{ENT}}_{{{\text{GA}}({\text{BeN}}_{4} )}}$, ${\text{ENT}}_{{{\text{M}}_{1} ({\text{BeN}}_{4} )}}$ and ${\text{ENT}}_{{{\text{M}}_{2} ({\text{BeN}}_{4} )}}$.
- **Harmonic zagreb entropy**
  $$\begin{aligned}  {\text{ENT}}_{{{\text{HM}}({\text{BeN}}_{4} )}}  &=\log (HM)-{\frac{1}{(HM)}}\sum _{i=1}^{6}\Theta {(\rho +\varrho )^2}\log _{2}{(\rho +\varrho )^2}\\&=\log (340mn-96m-208n+64)-\frac{(2)\log (9)^9}{340mn-96m-208n+64}-\frac{(2)\log (16)^{16}}{340mn-96m-208n+64}\\&\quad -\frac{(2n-2)\log (16)^{16}}{340mn-96m-208n+64}-\frac{(4m+4n-6)\log (25)^{25}}{340mn-96m-208n+64}-\frac{(4mn-3n)\log (36)^36}{340mn-96m-208n+64}\\&\quad -\frac{(4mn-4m-4n+4)\log (49)^49}{340mn-96m-208n+64}\\ \end{aligned}$$
- **Forgotton entropy**
  $$\begin{aligned}  {\text{ENT}}_{{{\text{F}}({\text{BeN}}_{4} )}}  &=\log (F)-{\frac{1}{(F)}}\sum _{i=1}^{6}\Theta {(\rho ^2+\varrho ^2)}\log _{2}{(\rho ^2+\varrho ^2)}\\&=\log (172mn-48m-104n+36)-\frac{(2)\log (4)^4}{172mn-48m-104n+36}-\frac{(2)\log (10)^{10}}{172mn-48m-104n+36}\\&\quad -\frac{(2n-2)\log (8)^8}{172mn-48m-104n+36}-\frac{(4m+4n-6)\log (13)^{13}}{172mn-48m-104n+36}-\frac{(4mn-3n)\log (18)^{18}}{172mn-48m-104n+36}\\&\quad -\frac{(4mn-4m-4n+4)\log (25)^{25}}{172mn-48m-104n+36}\\ \end{aligned}$$
- **Augmented zagreb entropy**
  $$\begin{aligned}  {\text{ENT}}_{{{\text{AZI}}({\text{BeN}}_{4} )}}  &=\log (AZI)-{\frac{1}{(AZI)}}\sum _{i=1}^{6}\Theta {\left( \frac{\rho \times \varrho }{\rho +\varrho -2}\right) ^3}\log _{2}{\left( \frac{\rho \times \varrho }{\rho +\varrho -2}\right) ^3}\\&=\log (100.8585mn-23.2960m-52.8585n+14.0460)\\{} & {} -\frac{(2)\log ((\frac{2}{1})^3)^{(\frac{2}{1})^3}}{100.8585mn-23.2960m-52.8585n+14.0460}\\{} & {} -\frac{(2)\log ((\frac{3}{2})^3)^{(\frac{3}{2})^3}}{100.8585mn-23.2960m-52.8585n+14.0460}\\{} & {} -\frac{(2n-2)\log ((\frac{4}{2})^3)^{(\frac{4}{2})^3}}{100.8585mn-23.2960m-52.8585n+14.0460}\\{} & {} -\frac{(4m+4n-6)\log ((\frac{6}{3})^3)^{(\frac{6}{3})^3}}{100.8585mn-23.2960m-52.8585n+14.0460}\\{} & {} -\frac{(4mn-3n)\log ((\frac{9}{4})^3)^{(\frac{9}{4})^3}}{100.8585mn-23.2960m-52.8585n+14.0460}\\{} & {} -\frac{(4mn-4m-4n+4)\log ((\frac{12}{5})^3)^{(\frac{12}{5})^3}}{100.8585mn-23.2960m-52.8585n+14.0460}\\ \end{aligned}$$
- **First redefined zagreb entropy**
  $$\begin{aligned}  {\text{ENT}}_{{{\text{ReZG}}_{1} ({\text{BeN}}_{4} )}}  &=\log (ReZG_1)-{\frac{1}{(ReZG_1)}}\sum _{i=1}^{6}\Theta {\left( \frac{\rho +\varrho }{\rho \times \varrho }\right) }\log _{2}{\left( \frac{\rho +\varrho }{\rho \times \varrho }\right) }\\ ReZG_1&=\log (5mn+m+0.3333n+1)-\frac{(2)\log (\frac{3}{2})^{\frac{3}{2}}}{5mn+m+0.3333n+1}\\{} & {} -\frac{(2)\log (\frac{4}{3})^{\frac{4}{3}}}{5mn+m+0.3333n+1}\\{} & {} -\frac{(2n-2)\log (\frac{4}{4})^{\frac{4}{4}}}{5mn+m+0.3333n+1}-\frac{(4m+4n-6)\log (\frac{5}{6})^{\frac{5}{6}}}{5mn+m+0.3333n+1}\\{} & {} -\frac{(4mn-3n)\log (\frac{6}{9})^{\frac{6}{9}}}{5mn+m+0.3333n+1}-\frac{(4mn-4m-4n+4)\log (\frac{7}{12})^{\frac{7}{12}}}{5mn+m+0.3333n+1}\\ \end{aligned}$$
  The numerical and graphical representation of ${\text{ENT}}_{{{\text{HM}}({\text{BeN}}_{4} )}}$, ${\text{ENT}}_{{{\text{F}}({\text{BeN}}_{4} )}}$, ${\text{ENT}}_{{{\text{AZI}}({\text{BeN}}_{4} )}}$ and ${\text{ENT}}_{{{\text{ReZG}}_{1} ({\text{BeN}}_{4} )}}$ is shown in Table 10 and Fig. 8, respectively.

**Table 10:** The numerical representation of ${\text{ENT}}_{{{\text{HM}}({\text{BeN}}_{4} )}}$, ${\text{ENT}}_{{{\text{F}}({\text{BeN}}_{4} )}}$, ${\text{ENT}}_{{{\text{AZI}}({\text{BeN}}_{4} )}}$ and ${\text{ENT}}_{{{\text{ReZG}}_{1} ({\text{BeN}}_{4} )}}$.

[*m*, *n*]	[1, 1]	[2, 2]	[3, 3]	[4, 4]	[5, 5]	[6, 6]	[7, 7]	[8, 8]	[9, 9]	[10, 10]
${\text{ENT}}_{{{\text{HM}}({\text{BeN}}_{4} )}}$	1.713	3.2527	4.1295	4.7385	5.2051	5.5833	5.9013	6.17566	6.4168	6.6321
${\text{ENT}}_{{{\text{F}}({\text{BeN}}_{4} )}}$	1.7247	3.2617	4.1336	4.7406	5.2062	5.5838	5.9014	6.1755	6.4165	6.6316
${\text{ENT}}_{{{\text{AZI}}({\text{BeN}}_{4} )}}$	1.728	3.2874	4.1566	4.761	5.2246	5.6008	5.9174	6.1906	6.431	6.6455
${\text{ENT}}_{{{\text{ReZG}}_{1} ({\text{BeN}}_{4} )}}$	1.7633	3.2889	4.1574	4.7623	5.2265	5.6029	5.9197	6.193	6.4335	6.6481

**Figure 8:**
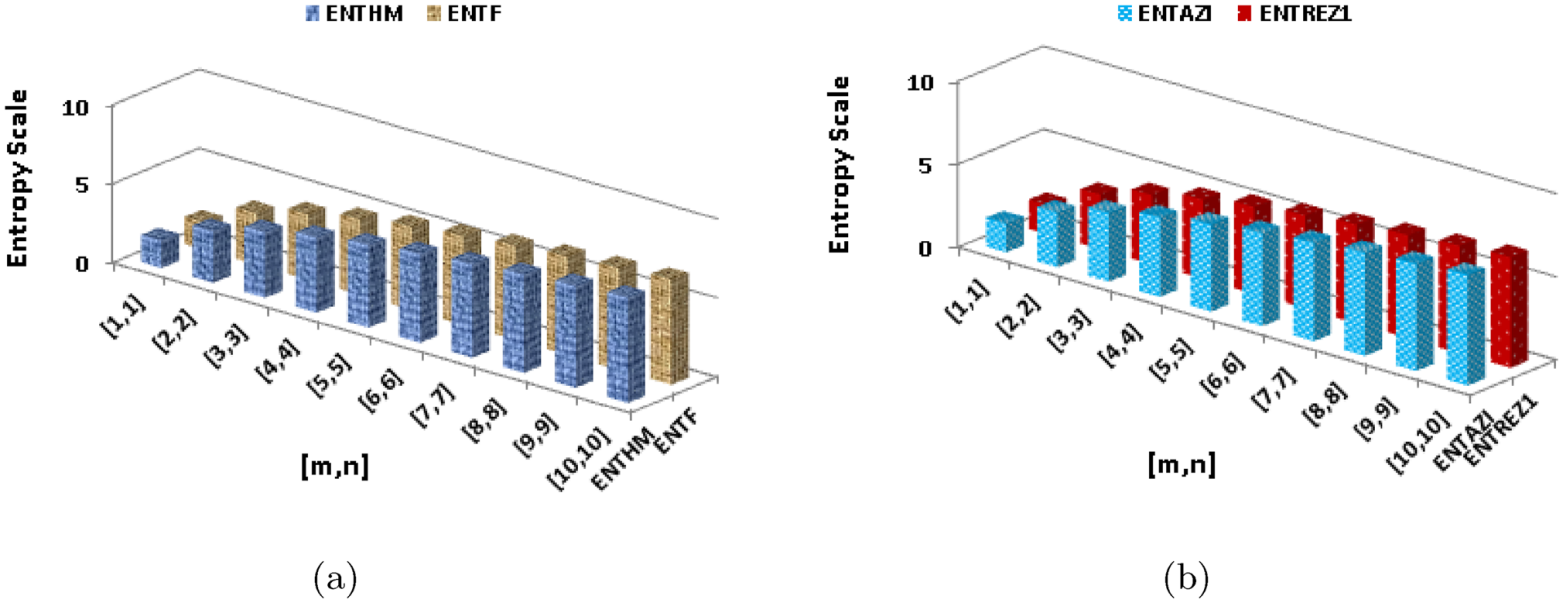
The numerical graphical representation of ${\text{ENT}}_{{{\text{HM}}({\text{BeN}}_{4} )}}$, ${\text{ENT}}_{{{\text{F}}({\text{BeN}}_{4} )}}$, ${\text{ENT}}_{{{\text{AZI}}({\text{BeN}}_{4} )}}$ and ${\text{ENT}}_{{{\text{ReZG}}_{1} ({\text{BeN}}_{4} )}}$.
- **Second redefined zagreb entropy**
  $$\begin{aligned}  {\text{ENT}}_{{{\text{ReZG}}_{2} ({\text{BeN}}_{4} )}}  &=\log (ReZG_2)-{\frac{1}{(ReZG_2)}}\sum _{i=1}^{6}\Theta {\left( \frac{\rho \times \varrho }{\rho +\varrho }\right) }\log _{2}{\left( \frac{\rho \times \varrho }{\rho +\varrho }\right) }\\&=\log (12.8571mn-2.0571m-6.0571n+0.4905)-\frac{(2)\log (\frac{2}{3})^{\frac{2}{3}}}{12.8571mn-2.0571m-6.0571n+0.4905}\\&\quad -\frac{(2)\log (\frac{3}{4})^{\frac{3}{4}}}{12.8571mn-2.0571m-6.0571n+0.4905}-\frac{(2n-2)\log (\frac{4}{4})^{\frac{4}{4}}}{12.8571mn-2.0571m-6.0571n+0.4905}\\&\quad -\frac{(4m+4n-6)\log (\frac{6}{5})^{\frac{6}{5}}}{12.8571mn-2.0571m-6.0571n+0.4905}-\frac{(4mn-3n)\log (\frac{9}{6})^{\frac{9}{6}}}{12.8571mn-2.0571m-6.0571n+0.4905}\\&\quad -\frac{(4mn-4m-4n+4)\log (\frac{12}{7})^{\frac{12}{7}}}{12.8571mn-2.0571m-6.0571n+0.4905}\\ \end{aligned}$$
- **Third redefined zagreb entropy**
  $$\begin{aligned}  {\text{ENT}}_{{{\text{ReZG}}_{3} ({\text{BeN}}_{4} )}}  &=\log (ReZG_3)-{\frac{1}{(ReZG_3)}}\sum _{i=1}^{6}\Theta {\left( ({\rho \times \varrho })({\rho +\varrho })\right) }\log _{2}{\left( ({\rho \times \varrho })({\rho +\varrho })\right) }\\&=\log (544mn-208m-392n+152)-\frac{(2)\log (6)^6}{544mn-208m-392n+152}-\frac{(2)\log (12)^{12}}{544mn-208m-392n+152}\\&\quad -\frac{(2n-2)\log (16)^{16}}{544mn-208m-392n+152}-\frac{(4m+4n-6)\log (30)^{30}}{544mn-208m-392n+152}-\frac{(4mn-3n)\log (54)^{54}}{544mn-208m-392n+152}\\&\quad -\frac{(4mn-4m-4n+4)\log (84)^{84}}{544mn-208m-392n+152}\\ \end{aligned}$$
  The numerical and graphical representation of ${\text{ENT}}_{{{\text{ReZG}}_{2} ({\text{BeN}}_{4} )}}$, and ${\text{ENT}}_{{{\text{ReZG}}_{3} ({\text{BeN}}_{4} )}}$ is shown in Table 11 and Fig. 9, respectively.

**Table 11:** The numerical representation of ${\text{ENT}}_{{{\text{ReZG}}_{2} ({\text{BeN}}_{4} )}}$, and ${\text{ENT}}_{{{\text{ReZG}}_{3} ({\text{BeN}}_{4} )}}$.

[*m*, *n*]	[1, 1]	[2, 2]	[3, 3]	[4, 4]	[5, 5]	[6, 6]	[7, 7]	[8, 8]	[9, 9]	[10, 10]
${\text{ENT}}_{{{\text{ReZG}}_{1} ({\text{BeN}}_{4} )}}$	1.7571	3.3009	4.1681	4.7709	5.2334	5.6087	5.9246	6.1973	6.4373	6.6515
${\text{ENT}}_{{{\text{ReZG}}_{1} ({\text{BeN}}_{4} )}}$	1.5934	3.1255	4.0156	4.6311	5.1015	5.4822	5.8019	6.0775	6.3198	6.5358

**Figure 9:**
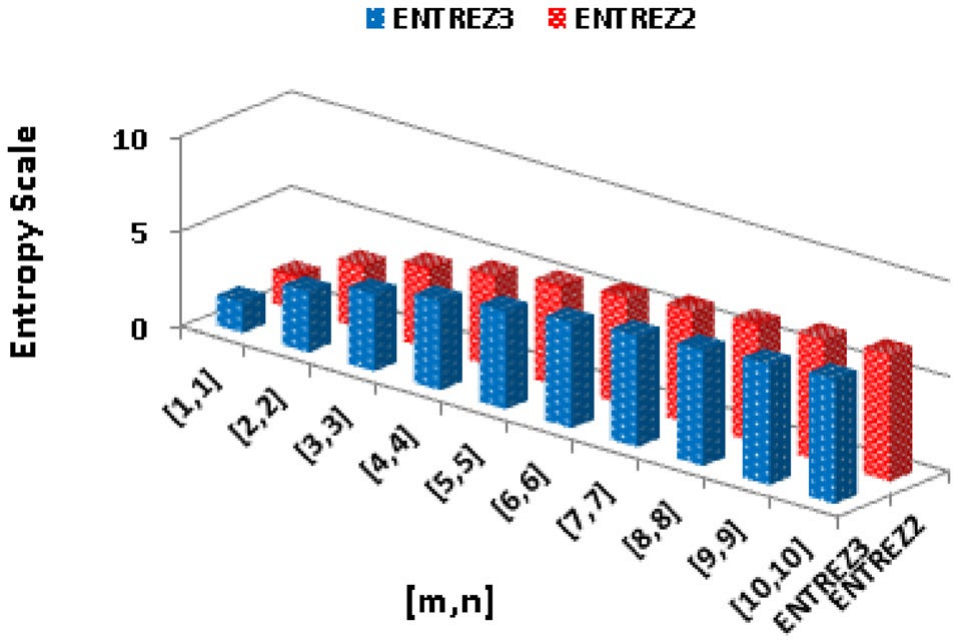
The graphical representation of ${\text{ENT}}_{{{\text{ReZG}}_{2} ({\text{BeN}}_{4} )}}$, and ${\text{ENT}}_{{{\text{ReZG}}_{3} ({\text{BeN}}_{4} )}}$.

## Logarithmic regression model and its analysis

A dependent variable and one or more independent variables are modeled, and the connection between them is examined using the statistical approach known as regression analysis^39^. It is frequently used to comprehend the effects of independent factors on the dependent variable and create forecasts or estimates in various domains, including economics, finance, social sciences, and engineering^40^. Regression analysis’ fundamental premise is to identify the line or curve that best captures the connection between the variables. The variable you seek to predict or explain is the dependent variable, called the response variable. The variables expected to impact the dependent variable are referred to as independent variables, often known as predictor variables or explanatory variables^41^. We used the SPSS software for these analysis (https://www.ibm.com/products/spss-statistics). Regression analysis may have many different forms, but the most popular one is basic linear regression, which only requires one independent variable. The relationship between the variables is considered linear in basic linear regression^42^. The line’s equation is displayed as:

$$\begin{aligned} Y = \beta _{0}+\beta _{1}X_{1}+\beta _{2}X_{2}+\beta _{3}X_{3}+ \cdots +\beta _{z}X_{z}+\varepsilon \end{aligned}$$

where, *Y* is the dependent variable, $\beta _{0}$ is the Y-intercept, $\beta _{i}$ is the Coefficients of independent variable for $i=1...z$, *X* is the Independent variable and, $\varepsilon$ is the Error.

To minimize the sum of squared differences between the observed values of *Y* and the anticipated values from the model, regression analysis aims to estimate the values of $\beta _{0}$ and $\beta _{1}$. The least squares method is commonly used for this estimating process. Regression analysis also offers several statistical measures to evaluate the model’s quality, such as the coefficient of determination $(R^2)$, which shows the percentage of the dependent variable’s variance that can be accounted for by the independent variables. Regression analysis is a potent tool for figuring out how variables relate to one another, formulating predictions, and investigating cause-and-effect relationships. It is widely used in many disciplines for data analysis, decision-making, and research^43^.

A statistical method for modeling the relationship between a dependent variable and one or more independent variables where a logarithmic scale may better represent the relationship is known as logarithmic regression analysis, logarithmic transformation, or log-linear regression^44^.

$$\begin{aligned} Y = \beta _{0}+\beta _{1}logX_{1}+\beta _{2}logX_{2}+\beta _{3}logX_{3}+ \cdots +\beta _{z}logX_{z}+\varepsilon \end{aligned}$$

where, *Y* is the dependent variable, $\beta _{0}$ is the Y-intercept, $\beta _{i}$ is the Coefficients of independent variable for $i=1 \ldots z$, *X* is the Independent variable, *log*() is the log function, and $\varepsilon$ is the Error.

The logarithmic transformation enables the modeling of relationships in which the independent variables’ effects on the dependent variable are multiplicative rather than additive. It is frequently employed when the relationship between the variables is curvilinear, with declining returns or increasing rates of change^45^. Logarithmic regression can be applied to data analysis in various domains, including economics, finance, biology, and environmental sciences^46^. It enables researchers to record and evaluate non-linear correlations between variables, as well as make predictions or draw insights using the logarithmic scale.

## Discussion on computed results

Using the *SPSS* software, basically two regression models (logarithmic and power) are applied to examine the relationship between $\text{TI}$ and graph entropy. It is noticed that the curve of logarithmic model is more closer then the power model because curve of logarithmic model touches almost each point of the observed data set, so we conclude that logarithmic model is more significant then the power, that is why logarithmic regression is applied to check the relationship between graph topological indices and entropy. The basic purpose of applying regression is to check the best predictor, the variable having good relation are the best predictor. In this case variables are curvilinear, so the best model to show their relationship is logarithmic regression.. As curve of logarithmic model passes through exactly each point of ${\text{GA}}({\text{BeN}}_{4} )$, so we may say that the relationship between ${\text{GA}}({\text{BeN}}_{4} )$ and its corresponding entropy ${\text{ENT}}_{{{\text{GA}}}} ({\text{G}})$ is much more better than the other $\text{TI}$. Here we use different symbols for indices and entropy in the Figures that are $R1=  {\text{R}}_{1}   ({\text{BeN}}_{4} )$, $RN1=  {\text{R}}_{-1}   ({\text{BeN}}_{4} )$, $R12= {\text{R}}_{{\frac{1}{2}}} ({\text{BeN}}_{4} )$, $RN12= {\text{R}}_{{-\frac{1}{2}}} ({\text{BeN}}_{4} )$, $ABC= {\text{ABC}}({\text{BeN}}_{4} )$, $GA = {\text{GA}}({\text{BeN}}_{4} )$, $M1= {\text{M}}_{1} ({\text{BeN}}_{4} )$, $M2= {\text{M}}_{2} ({\text{BeN}}_{4} )$, $HM= {\text{HM}}({\text{BeN}}_{4} )$, $F= {\text{F}}({\text{BeN}}_{4} )$, $REZ1= {\text{ReZG}}_{1} ({\text{BeN}}_{4} )$, $REZ2= {\text{ReZG}}_{2} ({\text{BeN}}_{4} )$ and, $REZ3= {\text{ReZG}}_{3} ({\text{BeN}}_{4} )$. Similarly, $ENTR1= {\text{ENT}}_{{ {\text{R}}_{1}   ({\text{BeN}}_{4} )}}$, $ENTRN1= {\text{ENT}}_{{{\text{R}}_{{ - 1}} ({\text{BeN}}_{4} )}}$, $ENTR12= {\text{ENT}}_{{{\text{R}}_{{\frac{1}{2}}} ({\text{BeN}}_{4} )}}$, $ENTRN12= {\text{ENT}}_{{{\text{R}}_{{ - \frac{1}{2}}} ({\text{BeN}}_{4} )}}$, $ENTABC= {\text{ENT}}_{{{\text{ABC}}({\text{BeN}}_{4} )}}$, $ENTGA= {\text{ENT}}_{{{\text{GA}}({\text{BeN}}_{4} )}}$, $ENTM1= {\text{ENT}}_{{{\text{M}}_{1} ({\text{BeN}}_{4} )}}$, $ENTM2={\text{ENT}}_{{{\text{M}}_{2} ({\text{BeN}}_{4} )}}$, $ENTHM= {\text{ENT}}_{{{\text{HM}}({\text{BeN}}_{4} )}}$, $ENTF= {\text{ENT}}_{{{\text{F}}({\text{BeN}}_{4} )}}$, $ENTREZ1= {\text{ENT}}_{{{\text{ReZG}}_{1} ({\text{BeN}}_{4} )}}$, $ENTREZ2={\text{ENT}}_{{{\text{ReZG}}_{2} ({\text{BeN}}_{4} )}}$ and, $ENTREZ3={\text{ENT}}_{{{\text{ReZG}}_{3} ({\text{BeN}}_{4} )}}$.

It can be seen that ${\text{GA}}({\text{BeN}}_{4} )$ and ${\text{ENT}}_{{{\text{GA}}({\text{BeN}}_{4} )}}$ has best relationship having $R=1$, $R^2=1$, $S_{E}=0.011$ and $F=186557:243$. A model with maximum value of *R*, $R^2$ and *F*, while minimum $S_{E}$ is best model. So we may conclude that ${\text{GA}}({\text{BeN}}_{4} )$ is the best predictor of complexity of $BeO_4$.

**Figure 10:**
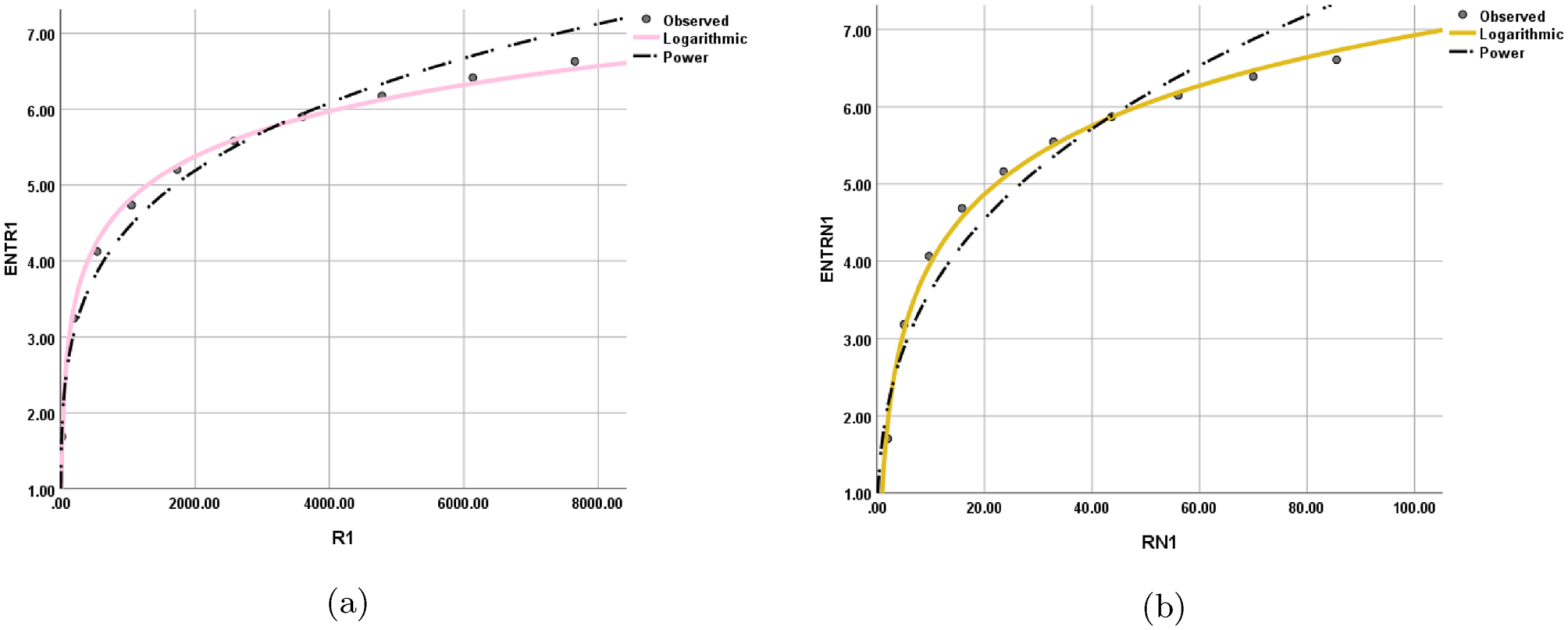
Logarithmic model between (**a**) ${\text{R}}_{1} ({\text{G}})$ and ${\text{ENT}}_{{{\text{R}}_{1} }} ({\text{G}})$, (**b**) ${\text{R}}_{-1} ({\text{G}})$ and ${\text{ENT}}_{{{\text{R}}_{-1} }} ({\text{G}})$.

**Figure 11:**
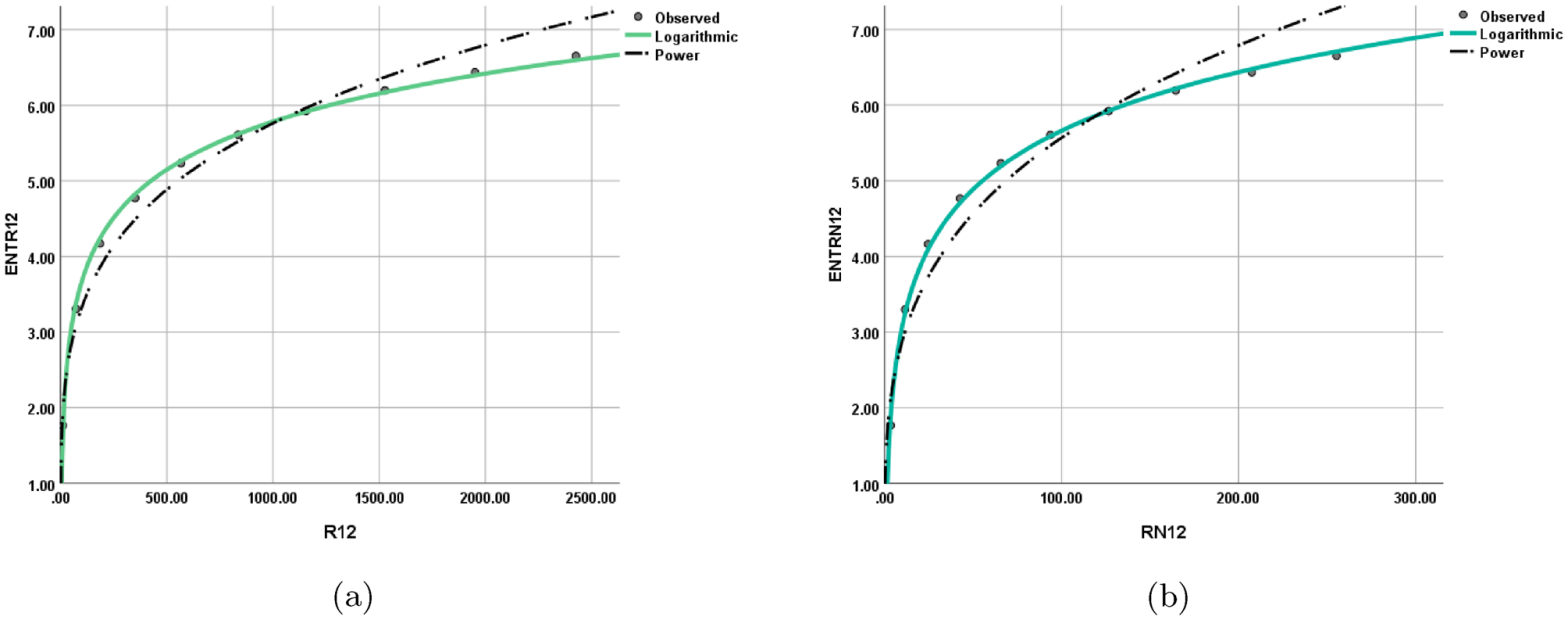
Logarithmic model between (**a**) ${\text{R}}_{{\frac{1}{2}}} ({\text{G}})$ and ${\text{ENT}}_{{{\text{R}}_{{\frac{1}{2}}} }} ({\text{G}})$, (**b**) ${\text{R}}_{{-\frac{1}{2}}} ({\text{G}})$ and ${\text{ENT}}_{{{\text{R}}_{{-\frac{1}{2}}} }} ({\text{G}})$.

**Figure 12:**
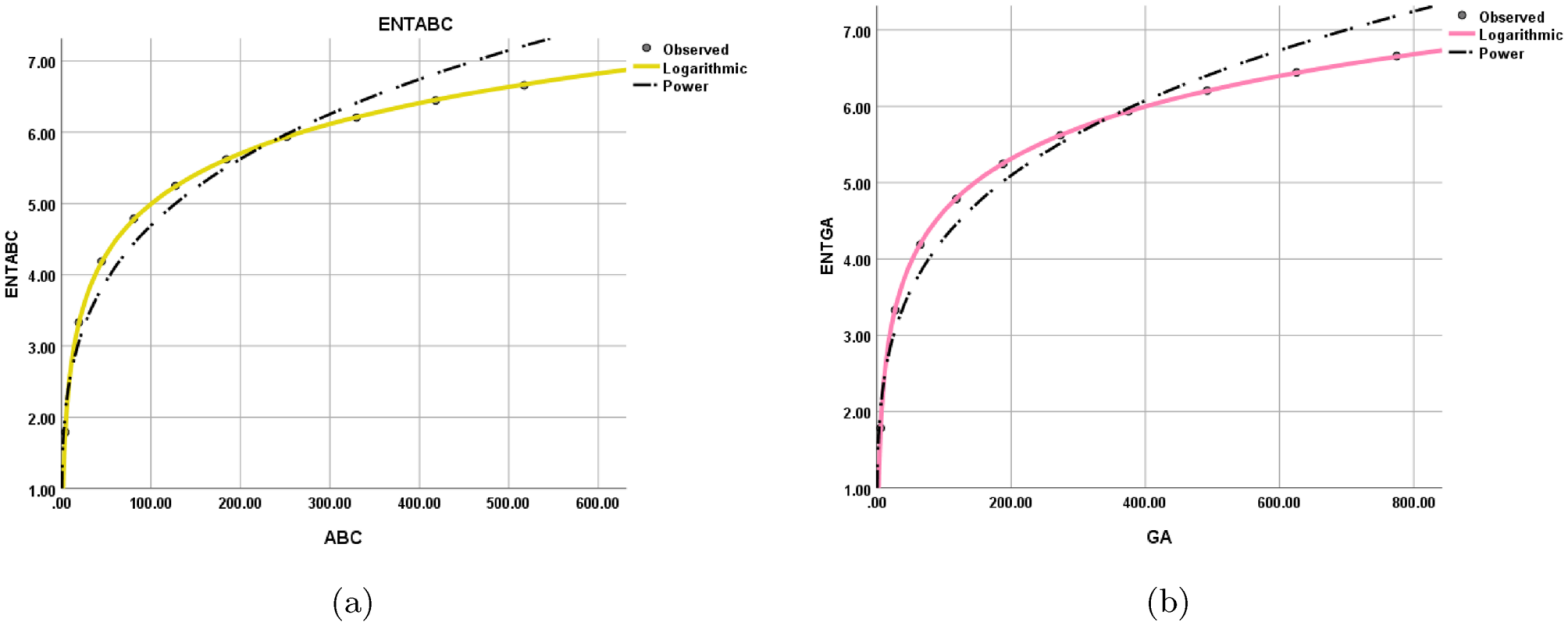
Logarithmic model between (**a**) $\text {ABC(G)}$ and ${\text{ENT}}_{{{\text{ABC}}}} ({\text{G}})$, (**b**) $\text {GA(G)}$ and ${\text{ENT}}_{{{\text{GA}}}} ({\text{G}})$.

**Figure 13:**
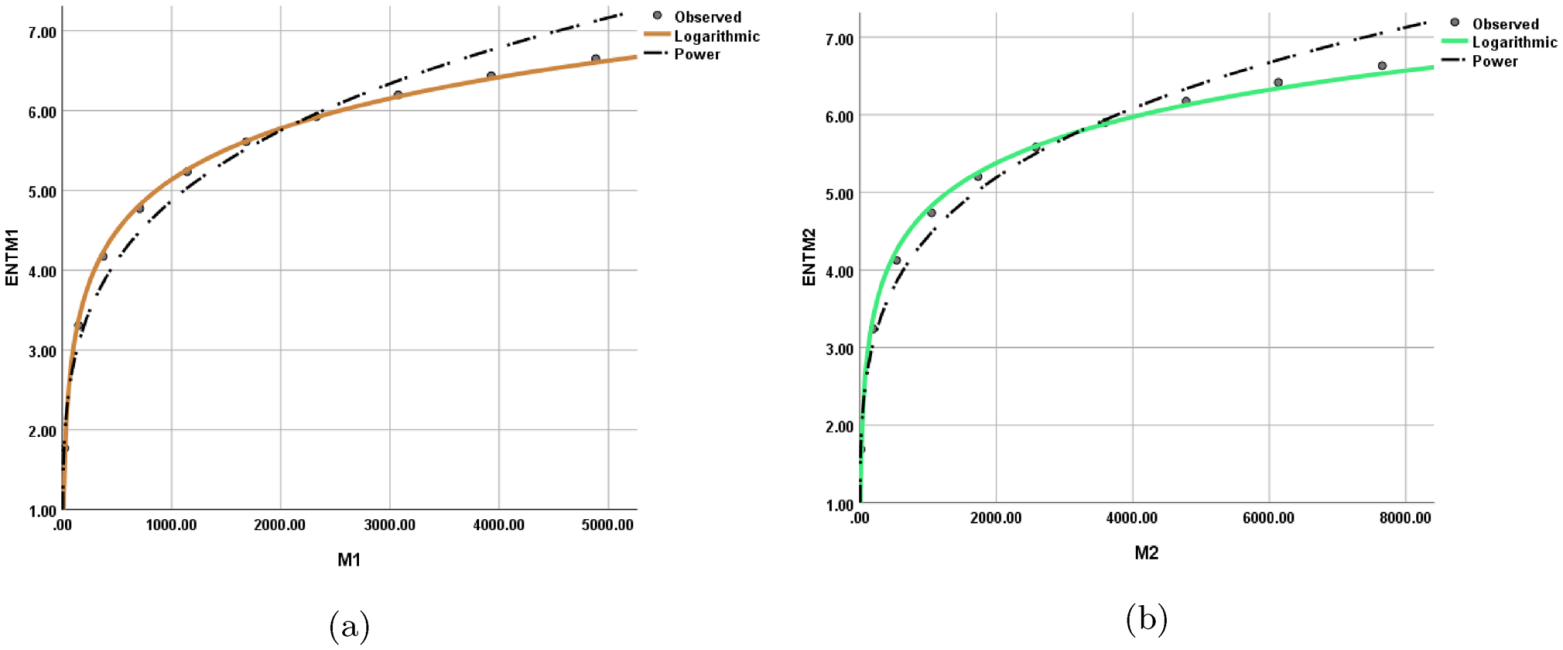
Logarithmic model between (**a**) ${\text{M}}_{1} ({\text{G}})$ and ${\text{ENT}}_{{{\text{M}}_{1} }} ({\text{G}})$, (**b**) ${\text{M}}_{2} ({\text{G}})$ and ${\text{ENT}}_{{{\text{M}}_{2} }} ({\text{G}})$.

**Figure 14:**
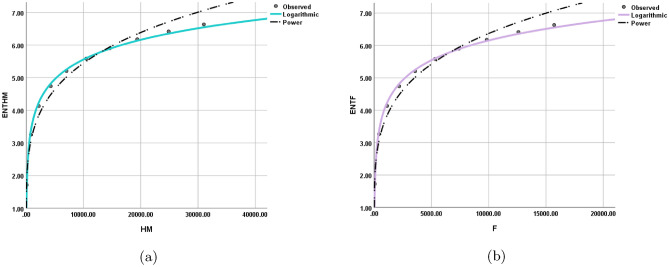
Logarithmic model between (**a**) $\text {HM(G)}$ and ${\text{ENT}}_{{{\text{HM}}}} ({\text{G}})$, (**b**) $\text {F(G)}$ and ${\text{ENT}}_{{\text{F}}} ({\text{G}})$.

**Figure 15:**
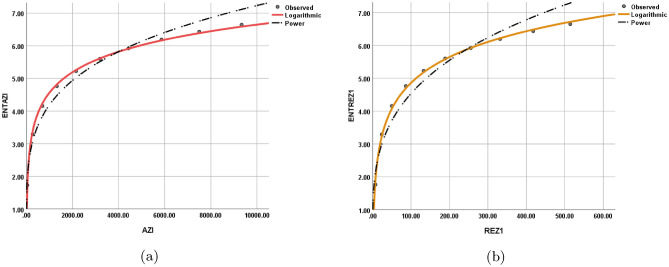
Logarithmic model between (**a**) $\text {AZI(G)}$ and ${\text{ENT}}_{{{\text{AZI}}}} ({\text{G}})$, (**b**) ${\text{ReZG}}_{1} ({\text{G}})$ and ${\text{ENT}}_{{{\text{ReZG}}_{1} }} ({\text{G}})$.

**Figure 16:**
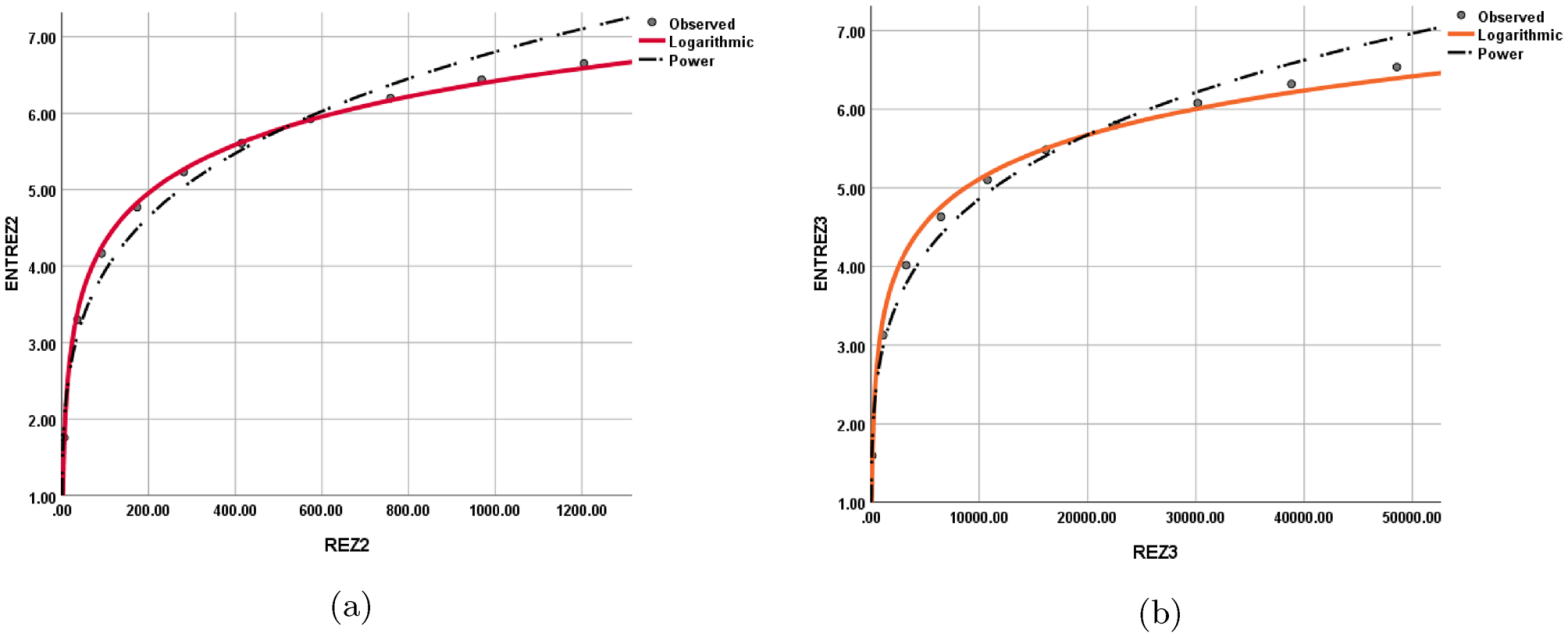
Logarithmic model between (**a**) ${\text{ReZG}}_{2} ({\text{G}})$ and ${\text{ENT}}_{{{\text{ReZG}}_{2} }} ({\text{G}})$, (**b**) ${\text{ReZG}}_{3} ({\text{G}})$ and ${\text{ENT}}_{{{\text{ReZG}}_{3} }} ({\text{G}})$.

The statistical values for each model are depicted in Tables 12, 13, 14, 15, 16, 17, 18, and 19 while the graphical depiction in the Figs. 10, 11, 12, 13, 14, 15, and 16.

**Table 12:** The statistical values for logarithmic model.

$Logarithmic\,\ Model$	*R*	$R^2$	$S_{E}$	*F*	*Significance*
$ENT_{{{\text{R}}_{1} }} ({\text{BeN}}_{4} ) = 0.86\ln [{\text{R}}_{1} ({\text{BeN}}_{4} )] - 1.158$	0.998	0.995	0.114	1689.506	0.000
$ENT_{{{\text{R}}_{{ - 1}} }} ({\text{BeN}}_{4} ) = 1.28\ln [{\text{R}}_{{ - 1}} ({\text{BeN}}_{4} )] + 1.032$	0.997	0.995	0.118	1560.834	0.000

**Table 13:** The statistical values for logarithmic model.

$Logarithmic\,\ Model$	*R*	$R^2$	$S_{E}$	*F*	*Significance*
$ENT_{{{\text{R}}_{{\frac{1}{2}}} }} ({\text{BeN}}_{4} ) = 0.918\ln [{\text{R}}_{{\frac{1}{2}}} ({\text{BeN}}_{4} )] - 0.554$	0.999	0.999	0.061	5839.737	0.000
$ENT_{ {\text{R}}_{{-\frac{1}{2}}} ({\text{BeN}}_{4} ) }=1.117\ln [ {\text{R}}_{{-\frac{1}{2}}} ({\text{BeN}}_{4} ) ]+0.519$	0.999	0.999	0.063	5453.919	0.000

**Table 14:** The statistical values for logarithmic model.

$Logarithmic\,\ model$	*R*	$R^2$	$S_{E}$	*F*	*Significance*
$ENT_{ {\text{ABC}}({\text{BeN}}_{4} ) }=1.022\ln [ {\text{ABC}}({\text{BeN}}_{4} ) ]+0.284$	1	1	0.013	123210.473	0.000
$ENT_{ {\text{GA}}({\text{BeN}}_{4} ) }=0.989\ln [ {\text{GA}}({\text{BeN}}_{4} ) ]+0.072$	1	1	0.011	186557.243	0.000

**Table 15:** The statistical values for logarithmic model.

$Logarithmic\,\ model$	*R*	$R^2$	$S_{E}$	*F*	*Significance*
$ENT_{ {\text{M}}_{1} ({\text{BeN}}_{4} ) }=0.926\ln [ {\text{M}}_{1} ({\text{BeN}}_{4} ) ]-1.259$	1	0.999	0.051	8238.997	0.000
$ENT_{ {\text{M}}_{2} ({\text{BeN}}_{4} ) }=0.86\ln [ {\text{M}}_{2} ({\text{BeN}}_{4} ) ]-1.158$	0.998	0.995	0.114	1689.506	0.000

**Table 16:** The statistical values for logarithmic model.

$Logarithmic\,\ Model$	*R*	$R^2$	$S_{E}$	*F*	*Significance*
$ENT_{ {\text{HM}}({\text{BeN}}_{4} ) }=0.871\ln [ {\text{HM}}({\text{BeN}}_{4} ) ]-2.461$	0.998	0.996	0.099	2217.52	0.000
$ENT_{ {\text{F}}({\text{BeN}}_{4} ) }=0.882\ln [ {\text{F}}({\text{BeN}}_{4} ) ]-1.965$	0.999	0.997	0.084	3052.608	0.000

**Table 17:** The statistical values for logarithmic model.

$Logarithmic\,\ Model$	*R*	$R^2$	$S_{E}$	*F*	*Significance*
$ENT_{ {\text{AZI}}({\text{BeN}}_{4} ) }=0.905\ln [ {\text{AZI}}({\text{BeN}}_{4} ) ]-1.692$	0.999	0.998	0.065	5229.637	0.000
$ENT_{ {\text{ReZG}}_{1} ({\text{BeN}}_{4} ) }=1.134\ln [ {\text{ReZG}}_{1} ({\text{BeN}}_{4} ) ]-0.362$	0.999	0.998	0.074	3923.561	0.000

**Table 18:** The statistical values for logarithmic model.

$Logarithmic\,\ Model$	*R*	$R^2$	$S_{E}$	*F*	*Significance*
$ENT_{ {\text{ReZG}}_{2} ({\text{BeN}}_{4} ) }=0.91\ln [ {\text{ReZG}}_{2} ({\text{BeN}}_{4} ) ]+0.133$	0.999	0.998	0.07	4474.447	0.000
$ENT_{ {\text{ReZG}}_{3} ({\text{BeN}}_{4} ) }=0.812\ln [ {\text{ReZG}}_{3} ({\text{BeN}}_{4} ) ]-2.367$	0.995	0.991	0.159	872.398	0.000

**Table 19:** The Goodness values for logarithmic model.

*Entropy*	$\beta _{1}$	$\beta _{0}$	*R*	$R^2$	$S_{E}$	*F*	*Significance*
$ENT_{R_{1}(BeN_4)}$	0.86	$-1.158$	0.998	0.995	0.114	1689.506	0.000
$ENT_{R_{-1}(BeN_4)}$	1.28	1.032	0.997	0.995	0.118	1560.834	0.000
$ENT_{R_{\frac{1}{2}}(BeN_4)}$	0.918	$-0.554$	0.999	0.999	0.061	5839.737	0.000
$ENT_{R_{-\frac{1}{2}}(BeN_4)}$	1.117	0.519	0.999	0.999	0.063	5453.919	0.000
$ENT_{ABC(BeN_4)}$	1.022	0.284	1	1	0.013	123210.473	0.000
$ENT_{GA(BeN_4)}$	0.989	0.072	1	1	0.011	186557.243	0.000
$ENT_{M_1(BeN_4)}$	0.926	$-1.259$	1	0.999	0.051	8238.997	0.000
$ENT_{M_2(BeN_4)}$	0.86	$-1.158$	0.998	0.995	0.114	1689.506	0.000
$ENT_{HM(BeN_4)}$	0.871	$-2.461$	0.998	0.996	0.099	2217.52	0.000
$ENT_{F(BeN_4)}$	0.882	$-1.965$	0.999	0.997	0.084	3052.608	0.000
$ENT_{AZI(BeN_4)}$	0.905	$-1.692$	0.999	0.998	0.065	5229.637	0.000
$ENT_{ReZG_1(BeN_4)}$	1.134	$-0.362$	0.999	0.998	0.074	3923.561	0.000
$ENT_{ReZG_2(BeN_4)}$	0.91	0.133	0.999	0.998	0.07	4474.447	0.000
$ENT_{ReZG_3(BeN_4)}$	0.812	$-2.367$	0.995	0.991	0.159	872.398	0.000

## Conclusion

This study delved into the intricate realm of Beryllonitrene’s molecular structure through the lens of graph theory and mathematical modeling. The computation and analysis of topological indices and graph entropy have illuminated crucial insights into the compound’s unique structural and energetic attributes. By employing logarithmic regression models, we established meaningful correlations between these indices, entropy, and other molecular characteristics, offering a comprehensive perspective on Beryllonitrene’s complex properties.

The findings underscore the significance of computational methodologies in deciphering the properties of novel materials, such as Beryllonitrene, which holds promise for diverse applications. The successful application of logarithmic regression models showcases their utility in capturing nuanced relationships within complex systems. Furthermore, the insights gained from this study provide a valuable foundation for potential applications of Beryllonitrene in various scientific and technological domains.As we move forward, this research sets the stage for further investigations into the molecular properties of Beryllonitrene and similar compounds. Additionally, the methodologies employed here could be extended to the analysis of other novel materials, contributing to the advancement of materials science and fostering innovation across disciplines. Ultimately, the integration of computational techniques and mathematical models in this study serves as a testament to their pivotal role in unraveling the mysteries of emerging materials and compounds.

The degree-based topological indices $\text{TI}$ are determined, as well as the entropy of graph based on these $\text{TI}$ to the complexity of $BeN_4$. It is noticed that by increasing the number of unit cell of $BeN_4$ the value of $\text{TI}$ and its corresponding entropy is also increasing which shows that as number of unit cell increases complexity of the $BeN_4$ also increases. Using the *SPSS* software, logarithmic and power regression is applied to examine the relationship between $\text{TI}$ and graph entropy. It is noticed that the line of logarithmic model is more closer then the power model because curve of logarithmic model touches almost each point of the observed data set so we conclude that logarithmic model is more significant then the power. As curve of logarithmic model passes through exactly each point of ${\text{GA}}({\text{BeN}}_{4} )$, so we may say that the relationship between ${\text{GA}}({\text{BeN}}_{4} )$ and its corresponding entropy ${\text{ENT}}_{{{\text{GA}}}} ({\text{G}})$ is much more better than the other $\text{TI}$ e.g (${\text{R}}_{1}   ({\text{BeN}}_{4} )$, ${\text{R}}_{-1}   ({\text{BeN}}_{4} )$, ${\text{R}}_{{\frac{1}{2}}} ({\text{BeN}}_{4}$, ${\text{R}}_{{ - \frac{1}{2}}} ({\text{BeN}}_{4} )$, ${\text{ABC}}({\text{BeN}}_{4} )$, ${\text{AZI}}({\text{BeN}}_{4} )$, ${\text{M}}_{1} ({\text{BeN}}_{4} )$, ${\text{M}}_{2} ({\text{BeN}}_{4} )$, ${\text{HM}}({\text{BeN}}_{4} )$, ${\text{F}}({\text{BeN}}_{4} )$, ${\text{ReZG}}_{1} ({\text{BeN}}_{4} )$, ${\text{ReZG}}_{2} ({\text{BeN}}_{4} )$, and ${\text{ReZG}}_{3} ({\text{BeN}}_{4} )$) because it has highest value of $R=1$, $R^2=1$ and $F=186557:243$, while the vale of $S_{E}=0.011$ is minimum as compared to the other $\text{TI}$. So we may conclude that ${\text{GA}}({\text{BeN}}_{4} )$ is best predictor of complexity $BeN_4$ of among all these indices.

## Data Availability

The datasets used and/or analysed during the current study available from the corresponding author on reasonable request.
